# The transcription factor LHX2 mediates and enhances oncogenic BMP signaling in medulloblastoma

**DOI:** 10.1038/s41418-025-01488-6

**Published:** 2025-03-28

**Authors:** Yae Ohata, Mohamad M. Ali, Yutaro Tsubakihara, Yuka Itoh, Gabriela Rosén, Tobias Bergström, Anita Morén, Irene Golán-Cancela, Ayana Nakada, Oleksandr Voytyuk, Maiko Tsuchiya, Rei Fukui, Kouhei Yamamoto, Paula Martín-Rubio, Patricia Sancho, Carina Strell, Patrick Micke, Robert J. Wechsler-Reya, Yoshinobu Hashizume, Kohei Miyazono, Laia Caja, Carl-Henrik Heldin, Fredrik J. Swartling, Aristidis Moustakas

**Affiliations:** 1 Department of Medical Biochemistry and Microbiology, Science for Life Laboratory, Box 582, Biomedical Center, Uppsala University, Uppsala, Sweden; 2 Department of Biochemistry, Graduate School of Medicine, University of Yamanashi, Chuo, Yamanashi Japan; 3 Department of Immunology, Genetics and Pathology, Rudbeck Laboratory, Science for Life Laboratory, Uppsala University, Uppsala, Sweden; 4 Faculty of Pharmaceutical Science, The University of Tokyo, Bunkyo-ku, Tokyo Japan; 5 Department of Oral Pathology, Tokyo Medical and Dental University, Bunkyo-ku, Tokyo Japan; 6 Department of Pathology, Teikyo University School of Medicine, Itabashi-ku, Tokyo Japan; 7 Department of Pathology, Nihon University School of Dentistry, Chiyoda-ku, Tokyo Japan; 8 Department of Comprehensive Pathology, Graduate School of Medical and Dental Sciences, Tokyo Medical and Dental University, Tokyo, Japan; 9 Department of Human Pathology, Graduate School of Medical and Dental Sciences, Tokyo Medical and Dental University, Tokyo, Japan; 10 Translational Research Unit, Hospital Universitario Miguel Servet, IIS Aragon, Zaragoza, Spain; 11 Center for Cancer Biomarkers (CCBIO), Department of Clinical Medicine, University of Bergen, Bergen, Norway; 12 Herbert Irving Comprehensive Cancer Center, Columbia University Medical Center, New York, NY USA; 13 RIKEN Program for Drug Discovery and Medical Technology Platforms, Wako, Saitama Japan; 14 Department of Applied Pathology, Graduate School of Medicine, The University of Tokyo, Bunkyo-ku, Tokyo Japan

**Keywords:** Paediatric cancer, Cancer microenvironment

## Abstract

Oncogenic events perturb cerebellar development leading to medulloblastoma, a common childhood brain malignancy. Molecular analyses classify medulloblastoma into the WNT, SHH, Group 3 and Group 4 subgroups. Bone morphogenetic protein (BMP) pathways control cerebellar development and have been linked to the progression of medulloblastoma disease, with major remaining gaps in their mechanistic and clinically-relevant roles. We therefore aimed at exploring BMP mechanisms of action in medulloblastoma. Patient-derived tumors from different subgroups were analyzed in mouse xenografts, complemented by independent tumor immunohistochemical analysis. Cell-based assays analyzed signaling mechanisms. Medulloblastoma cell orthotopic xenografts analyzed tumor growth and metastasis in vivo. Active BMP signaling, detected as nuclear and phosphorylated SMAD1/5, characterized several medulloblastoma subgroups, with enrichment in Group 4, SHH and Group 3 tumors. Spatial transcriptomics in tumor areas, complemented by transcriptomic analysis of multiple cell models, identified BMP-dependent transcriptional induction of the *LIM-homeobox gene 2* (*LHX2*). BMP signaling via SMADs induced *LHX2* expression and LHX2 transcriptionally induced BMP type I receptor (ACVR1) expression by association with the proximal promoter region of the *ACVR1* gene. BMP signaling and LHX2 gain-of-function expression led to enriched stemness and associated chemoresistance in medulloblastoma cultures. In-mouse orthotopic transplantation of paired primary/recurrent Group 4 medulloblastoma cell populations, correspondingly expressing LHX2-low/BMP-low signaling and LHX2-high/BMP-high signaling, ascribed to the latter (high) group more efficient tumor propagation and spinal cord metastatic potential. Depletion of LHX2 in these recurrent tumor cells suppressed both BMP signaling and tumor propagation in vivo. Thus, LHX2 cooperates with, and enhances, oncogenic BMP signaling in medulloblastoma tumors. The molecular pathway that couples LHX2 function to BMP signaling in medulloblastoma deepens our understanding this malignancy.

## Introduction

Medulloblastoma (MB), a childhood brain malignancy, develops when cerebellar progenitor cells undergo oncogenesis [1–4]. MB classification into four subgroups is based on clinical and molecular features identified by patient cohort analyses using genomics, DNA methylomics, transcriptomics, and proteomics. Wingless (WNT) MB, with aberrant β-catenin signaling -among other defect-s in ~15% of patients, have mossy fiber neurons of the lower rhombic lip as cell-of-origin [2, 5–9]. MB with mutations activating Sonic hedgehog (SHH) signaling, *MYCN* and *TP53* mutations in ~25% of the patients, have external granular layer neuronal progenitors as cell-of-origin [2, 5–9]. The most common (~60%) Group 3 and Group 4 MB, exhibiting complex molecular signatures, have progenitor cells or nascent unipolar brush cells located respectively in the rhombic lip’s ventricular or subventricular zones acting as cells-of-origin for Group 3, and nascent unipolar brush cells located in the rhombic lip subventricular zone for Group 4 [2, 5–9]. The oncogenic progenitors cannot complete differentiation and exhibit characteristic epigenetic and high-order chromatin features [6–9]. Moreover, *MYCN* amplifications, are misexpressed in Groups 3/4 or SHH MB [2, 3, 10, 11]. MYC-driven MB presents overactive pyrimidine synthesis, protein folding misregulation, therapeutic resistance and tumor recurrence [12–14]. Regulators of mRNA splicing and histone modifications operate oncogenically in distinct MB groups [15, 16]. Genome-wide chromatin immunoprecipitation-sequencing (ChIP-seq) analysis has revealed enhancer networks corresponding to distinct MB subgroups [17]. In Group 3/4 MB, the circuit of HLX, EOMES, LHX2, and LMX1A transcription factors has been proposed to generate positive feed-forward loops that regulate expression of each other and of downstream target genes [17].

Upstream of such epigenetic and transcriptional circuits lie developmental signaling pathways (e.g., WNT and SHH), and although not clearly delineated, the transforming growth factor β (TGFβ) and bone morphogenetic protein (BMP) families may also contribute based on their actions in cerebellar development. BMP family ligands signal via serine/threonine kinase receptors, which phosphorylate SMAD proteins (SMAD1, SMAD5, and SMAD8) and activate alternative signaling mediators, causing coordinate activation of SMAD and MAP-kinase pathways that regulate gene expression or mediate cytoplasmic signaling that controls cell differentiation [18].

Differentiation of the granule neurons, the most abundant cerebellar neurons, is induced by Bmp6 and Bmp7 in the mouse rhombic lip [19], an equivalent brain region where human Group 3/4 MB progenitors reside [6, 7, 9]. At such early developmental stages, BMPs induce expression of the transcription factor Atoh1/Math1, required for granule neuron specification [20]. In contrast, mouse Bmp2 and Bmp4 acting at a later stage of granular neuron development, induce proteolytic degradation of Atoh1/Math1, possibly explaining why BMPs induce cell death in certain MB cell cultures [21, 22]. At this developmental stage, Bmp2 and Bmp4 also suppress the SHH pathway, the major proliferative engine of granular neuron progenitors [23]. In agreement with a pro-differentiating action of BMP signaling, genes encoding BMP ligands or signaling mediators are epigenetically repressed in MB via DNA methylation or via the polycomb repressive complex protein BMI1 [22, 24, 25]. In contrast, Group 3 MBs with *MYCN* amplifications express BMP7, and a BMP receptor inhibitor suppresses the proliferation of MB cell cultures [26]. ATOH1/MATH1 can be inhibited by ID proteins, whose expression is potently induced by BMPs in every tissue [27], suggesting an oncogenic role for BMP signaling in MB. Thus, BMP signaling takes distinct and even opposite functional roles in MB [3].

The duality of BMP action in MB motivated this study. We here report on the cooperative oncogenic activity of the transcription factor LIM-homeobox gene 2 (LHX2) with BMP signaling. Comparative analyses of in vivo PDX (patient-derived xenograft) models and cell-based signaling models showed that LHX2 is induced by BMPs and enhances pro-oncogenic BMP activities in a feed-forward loop in MB.

## Methods

### Reagents, cells, viral infections, transfections, and cell-based assays

We have previously reported on the used reagents, MB cultures, lentiviral and adenoviral infections, siRNA and plasmid transfection protocols followed by cell-based assays (luciferase, viability, ELDA) [28–32]. The “Supplementary Materials and Methods” present details.

### Immunological techniques

Formalin-fixed, paraffin-embedded tumor samples were analyzed and immunoblotting was performed as described [28], with details in the Supplementary Methods and Supplementary Table S1 (primary antibodies). Original immunoblots are presented as supplementary files.

### RNA- and DNA-based analysis

Two PDXs were analyzed by NanoString GeoMx^®^ Digital Spatial Profiler, according to the manufacturer’s protocol (NanoString Technologies Inc., Seattle, WA, USA). Total cellular RNA-sequencing, CUT&RUN assays, and R2 data analyses were performed as described [31]. Details are in the Supplementary Methods, Supplementary Tables S2, S3 for GeoMx^®^, Supplementary Table S4 for cellular RNA-sequencing (primary data available at GEO - accession number GSE229150) and Supplementary Table S5 for PCR primer sequences.

### Quantification and statistical analysis

RT-qPCR and luciferase results represent mean values from at least three independent biological experiments. Each biological experiment included triplicate technical repeats. Error bars represent standard deviations (SD). The three biological replicates were used to determine average values and SD. For immunoblotting experiments, one representative result out of two to four independent repeats is presented. Statistical analysis was performed by Excel or R using two-tailed paired Student’s *t*-test or one-way ANOVA test followed by Dunnett’s multiple comparisons test as explained in the figure legends. Statistical significance was accepted when *p*-value < 0.05, and is indicated as: *p < 0.05, **p < 0.01, ***p < 0.001. Statistical analysis for the multiplex immunohistochemistry, spatial transcriptomic and RNA-seq experiments is described separately in the figure legends.

## Results

### Phosphorylated SMAD1/5 levels in different MB tumor subgroups

**Fig. 1:**
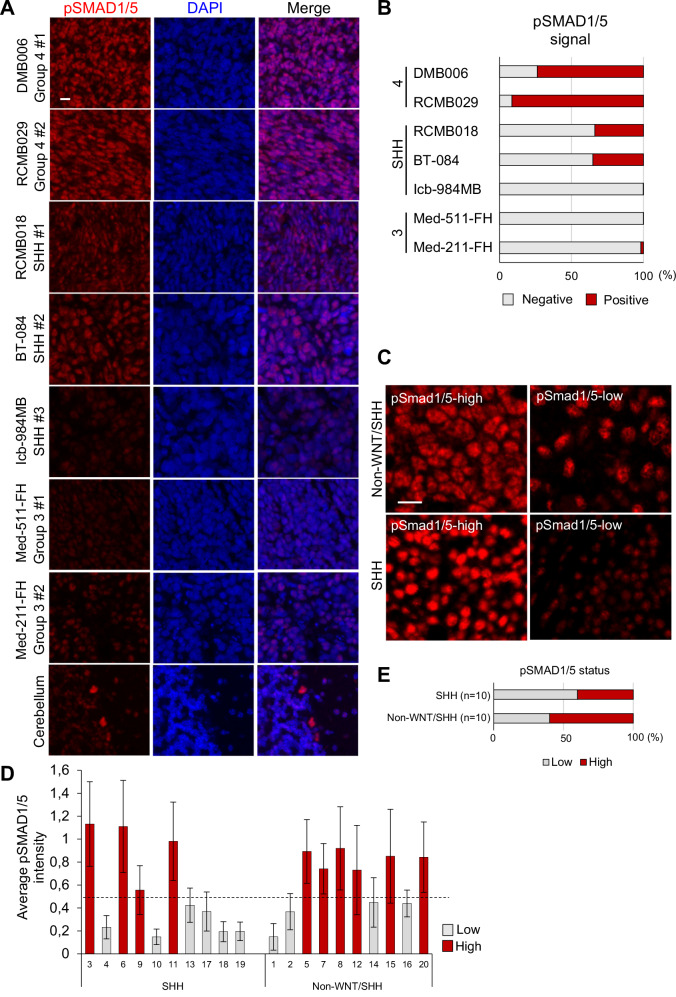
pSMAD1/5 multispectral immunofluorescence in PDX MB tumors. **A** Phosphorylated SMAD1/5 (pSMAD1/5) abundance, in seven medulloblastoma (MB) patient-derived xenografts (PDXs) in mice and in normal mouse cerebellum, was analyzed by multispectral immunofluorescence. Representative images are shown. Molecular subgroups are indicated under each PDX name. “#1” and “#2” indicate different PDX models. Scale bar: 10 μm. **B** Quantification of the pSMAD1/5 signals in the PDX MB sections, with representative images presented in **A**. Tumor cells were classified into two groups (negative or positive) based on the pSMAD1/5 staining intensities quantified in every cell in three selected areas per sample, representing a total of 4 000 to 10 000 cells, and plotted as percent of the total cell number. **C** Representative images of a human MB TMA highlighting SHH or non-WNT/SHH MB tumors immunostained for pSMAD1/5 as in panel (A). Scale bar: 10 μm. **D** Quantification of the pSMAD1/5 signals in each one of the 20 human MB TMA tumors, with representative images presented in **C**. Tumors were classified into two Groups (SHH or non-WNT/SHH) and pSMAD1/5 staining intensities are presented as low and high as in (**B**). The horizontal dotted line marks the median pSMAD1/5 intensity level. **E** Cumulative representation of the data in panel (D), quantified as in (**B**), except that pSMAD1/5 intensity is classified as low and high.

We analyzed seven PDX MB samples, three SHH, two Group 3 and two Group 4 (Fig. 1). Multispectral immunofluorescence analysis showed clear and quantifiable levels of C-terminally phosphorylated SMAD1/5 (pSMAD1/5), the universal marker of BMP signaling (Fig. 1A). Single cell-based quantification of pSMAD1/5 signals set a threshold across the analyzed tumors, classifying every single cell in representative areas as either pSMAD1/5-negative or -positive. This analysis revealed highest pSMAD1/5-positive scores in Group 4, intermediate signals in SHH and low signals in Group 3 samples (Fig. 1B). Since the available PDXs were few and variation of pSMAD1/5 scores could be observed (e.g., SHH PDXs, Fig. 1B), we also analyzed a commercially available TMA from MB patients, providing 20 tumor cores, half of them from SHH patients and the other half from Group 3/4 patients (WNT Group samples are rare and were not represented in this TMA) (Fig. 1C–E). Similar to the PDX analysis (Fig. 1A, B), the TMA tumors exhibited areas with strong and areas with weaker or very sparse pSMAD1/5 staining patterns (Fig. 1C). Upon quantification, 4 out of 10 SHH tumors resulted in high pSMAD1/5 scores, whereas 6 out of 10 of the non-WNT/SHH tumors (i.e., Group 3/4) gave high pSMAD1/5 scores (Fig. 1D, E). However, the limited number of PDX models and human tumor sections in the TMA does not allow us to reach a clear conclusion with respect to a significant difference in BMP-mediated pSMAD1/5 signals between Group 3 and Group 4 tumors.

We queried transcriptomic data of MB tumors and normal cerebellum [5, 33], using the R2 platform for genomic analysis and visualization (https://r2.amc.nl; Supplementary Fig. S1). By analyzing evolutionarily conserved and immediate-early *ID1*-*ID4* gene responses to BMP signaling [27], we found no prominent differences between subgroups, yet *ID3* and *ID4* mRNA levels were higher in MB relative to normal human cerebellum (Supplementary Fig. S1A). BMP receptor analysis identified high expression of *ACVR1*, *BMPR1A* and *BMPR2*, but not of *BMPR1B* mRNA, in all MB subgroups. Particularly, *ACVR1* expression was higher in Group 4 and 3, relative to SHH and WNT MB (Supplementary Fig. S1B). Among ligands, high *BMP4* expression relative to normal cerebellum was found only in WNT MB; all other subgroups and normal cerebellum showed strong expression of *BMP2*, *BMP6* and *BMP7* mRNA (Supplementary Fig. S1C). Further analysis revealed higher expression of *SMAD1* mRNA in SHH tumors and higher *SMAD5* mRNA expression in Group 3 and 4. On the other hand, the Z-score expression of a combined gene signature representing BMP signaling was significantly lower in SHH tumors compared to other MB subtypes (Supplementary Fig. S1D). We conclude that MB tumors, exhibiting clear patterns of pSMAD1/5 (Fig. 1), can also express specific BMP ligands, receptors, SMADs and downstream target genes, such as *ID3* and *ID4* (Supplementary Fig. S1). BMP signaling might be enriched in Group 4 or other MB subgroups (Fig. 1), a possibility worth investigating deeper.

### Spatial analysis reveals unique transcriptional profiles in pSMAD1/5-positive cells

**Fig. 2:**
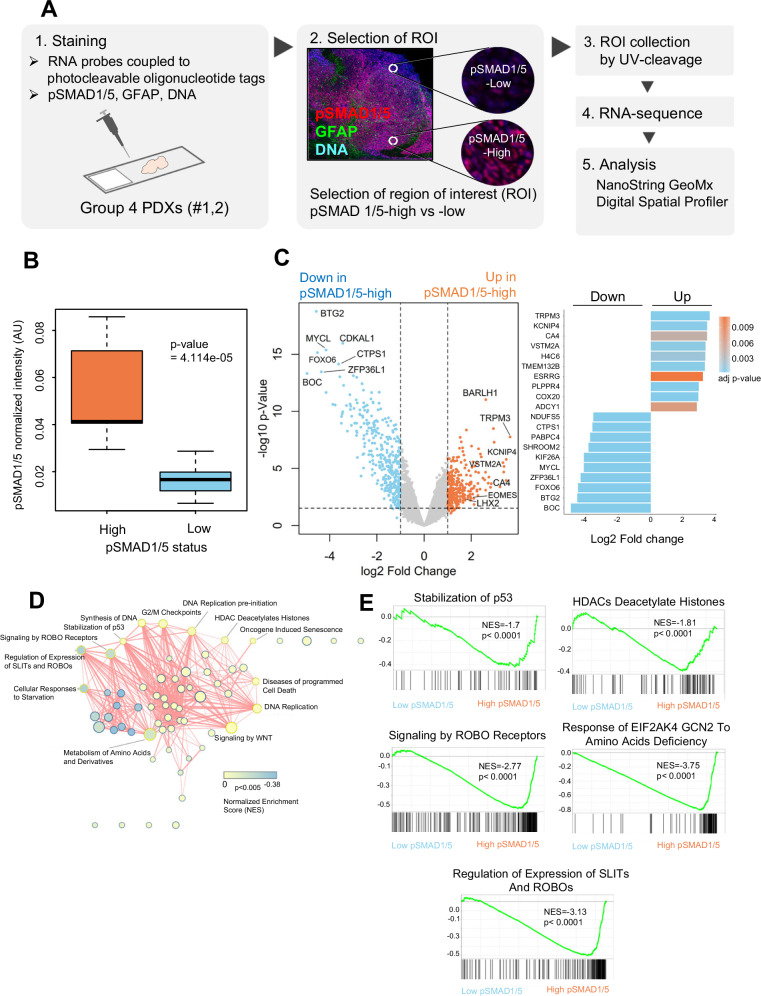
Spatial transcriptomic analysis of pSMAD1/5-high and -low tumor cells in Group 4 MB PDXs. **A** Schematic workflow of spatial RNA sequencing of MB PDXs performed by NanoString GeoMx^®^ Digital Spatial Profiler. Eighteen ROIs from the two Group 4 PDX samples (presented in Fig. 1 as Group 4 #1 and #2) with high or low phosphorylated SMAD1/5 (pSMAD1/5) levels were selected and subjected to analysis. **B** Box plot displaying the two pSMAD1/5 groups classified based on the median staining intensity of pSMAD1/5 (*n* = 18 ROIs, corresponding to 4000 to 10,000 cells per ROI). Horizontal lines indicate the median generated by quantification of more than 2000 data points, whiskers indicate the SEM and the *p*-value for the difference is also listed (derived using the Wilcoxon rank sum test). **C** Volcano plot of DEGs in pSMAD1/5-high cells with thresholds of significance shown as dotted lines (left). The top 10 up-regulated and top 10 down-regulated genes are listed (right). The color code represents the adjusted *p*-value. **D** Network analysis visualizing the interactions between significantly dysregulated pathways in pSMAD1/5-high tumor cells (*p*-value < 0.005 and overlap coefficient ≥ 0.5). Each node represents one biological process, and the size of the node corresponds to the number of the constituting genes. The lines refer to the significantly connected processes. The color code indicates the normalized enrichment score (NES). **E** Enrichment plots showing significantly regulated DEG sets in pSMAD1/5-high ROIs. Gene set enrichment analysis was performed using the Reactome database.

We then analyzed two Group 4 PDXs with the highest BMP signaling activity among the seven tumors studied (DMB006/Group 4#1 and RCMB029/Group 4#2; Fig. 1) in order to identify molecular pathways enriched in tumor cells presenting active BMP signaling in situ. We employed NanoString GeoMx® Digital Spatial Profiler for spatial transcriptomic analysis (Fig. 2A). Essentially all selected tumor cells were positive for pSMAD1/5. Yet, we divided the selected tumor sections into regions of interest (ROI) for the spatial analysis, nine with high and nine with low pSMAD1/5 intensity, in order to discriminate easier for differentially expressed genes (DEGs) linked to BMP signaling. To achieve this, pSMAD1/5 staining intensity was quantified in each selected ROI, and ROIs were classified based on the median normalized pSMAD1/5 signal (Fig. 2B). The mRNA expression of *SMAD6*, an immediate-early BMP target gene, was higher in pSMAD1/5-high cells, although with a statistically significant difference only in PDX#2; the ACVR1 and BMPR2 mRNAs showed a similar trend and were well expressed, similar to *ID1-4*, which were expressed in the ROIs of both tumors (Supplementary Fig. S2A). These data attest to the activation of BMP signaling in the selected ROIs.

Following spatial RNA sequencing analysis, we identified 570 DEGs (log_2_ fold-change ≥ ±1 and FDR < 0.05) between pSMAD1/5-high and -low ROIs (Fig. 2C; Supplementary Tables S2, S3). Among the upregulated DEGs, we noted several genes with established links to MB tumorigenesis (*BARLH1*, *TRPM3*, *KCNIP4*, *VSTM2A*, *CA4*, *EOMES* and *LHX2*; Fig. 2C). Correlation analysis of pSMAD1/5 signal intensity with the mRNA expression levels of 13 genes among the top DEGs indicated best correlation (0.67) with *LHX2* expression and correlations ranging between 0.51 (*PLPPR4*) and 0.02 (*BARHL1*) with the other DEGs (Supplementary Fig. S2B). Gene set enrichment analysis (GSEA) and visualization of network interaction revealed a significant downmodulation of cell cycle progression, histone deacetylation, amino acid metabolism, p53 stabilization, and ROBO/SLIT signaling, in pSMAD1/5-high compared to pSMAD1/5-low tumor cells (Fig. 2D, E; Supplementary Fig. S2C). The spatial analysis provided a rich resource of MB gene expression enriched in cells exhibiting BMP signaling, leaving the open question of whether these genes directly responded to BMP signaling.

### Comparative spatial and cellular RNA-sequencing analysis highlights LHX2

**Fig. 3:**
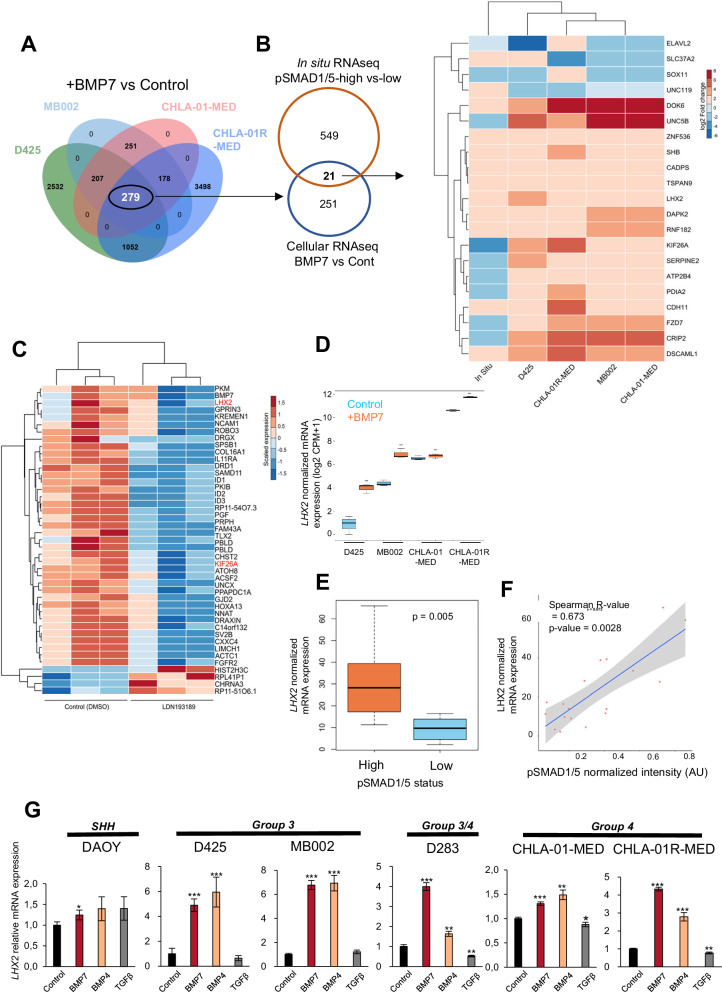
Comparative analysis of spatial and cellular RNA-seq identifies *LHX2.* **A** Four-way Venn diagram showing the numbers of DEGs in four MB cell lines (D425, MB002, CHLA-01-MED and CHLA-01R-MED) treated with BMP7 (100 ng/ml) for 3 days, relative to the respective untreated cells. The common 279 DEGs between the four cell lines are highlighted (white). **B** (Left) Venn diagram showing the number of DEGs shared between spatial RNA-seq analyzed in Fig. 2, and the 4 BMP7-treated cell lines shown in (A). The 28 common DEGs between the spatial and the cellular RNA-seq analysis are highlighted (bold). (Right) Heatmap visualizing expression levels of the common 28 DEGs in the 5 analyzed experimental conditions. The color-coded scale represents log_2_ fold change values of each transcript in the respective condition. **C** Heatmap visualizing expression levels of DEGs in CHLA-01R-MED cells treated with LDN193189 for 3 days relative to untreated cells. *LHX2* and *KIF26A* (highlighted in red) are common genes that were also highlighted in **B**. The color-coded scale represents the scaled counts per million (CPM) values of each transcript. **D** Box plot of normalized *LHX2* mRNA expression in the four cell lines analyzed by RNA-seq. Horizontal lines indicate the median, whiskers indicate the SEM and the *p*-value for the difference relative to control is listed ***p < 0.001 (derived using the Wilcoxon rank sum test). **E** Normalized *LHX2* mRNA expression in pSMAD1/5-high and -low tumor cells analyzed by spatial RNA-seq. Horizontal lines indicate the median, whiskers indicate the SEM and the significance level was derived using the Wilcoxon rank sum test. **F** Correlation plot showing a positive correlation between pSMAD1/5 signal intensity (arbitrary units, AU) and *LHX2* mRNA expression in 18 ROIs of the two Group 4 PDX tumors. The Spearman correlation value (*R*) and corresponding *p*-value are shown. **G** *LHX2* mRNA expression in MB cell lines was measured by RT-qPCR 3 days after treatment with BMP7 (100 ng/ml), BMP4 (100 ng/ml) or TGFβ1 (5 ng/ml). The results were normalized to *GAPDH* levels. Data are presented as mean values ± SD of three biological replicates. *p < 0.05, **p < 0.01, ***p < 0.001 com*p*ared with control (two-tailed paired Student’s *t* test).

We complemented the spatial analysis with RNA-seq analyses in four MB cell lines, two isogenic cell types, one derived from a primary Group 4 MB tumor (CHLA-01-MED) and the other from the respective metastasis of the primary tumor (CHLA-01R-MED) [34], as well as two Group 3 MB cell lines D425 and MB002. The cells were stimulated with BMP7, since it showed the highest relative expression in the majority of MB tumors (Supplementary Fig. S1C). Between 900 and 5000 DEGs were measured as up- or downregulated in response to BMP7 relative to the respective untreated cells (Fig. 3A; Supplementary Table S4). We focused on the 279 BMP7-responsive DEGs that were shared among the 4 cell lines (Fig. 3A). Comparing tumor and cellular transcriptomes revealed 28 DEGs commonly enriched in pSMAD1/5-high ROIs and BMP7-treated MB cells, some of which had very similar and some more distinct scaled expression between the five biological samples analyzed (Fig. 3B). Furthermore, we performed RNA-seq analysis in CHLA-01R-MED cells treated with the widely used BMP type I receptor kinase inhibitor LDN193189, aiming at scrutinizing genes that respond to autogenous BMP signaling (Fig. 3C; Supplementary Table S4). Among 43 DEGs, several hallmark targets of BMP signaling (*BMP7*, *SPSB1*, *SMAD11*, *ID1*, *ID2*, *ID3*, *TLX2*, *ATOH8*) were expressed in CHLA-01R-MED cells, and their expression was significantly repressed upon treatment with LDN193189 (Fig. 3C).

Integration of all RNA-seq datasets identified *LHX2*, among the 28 DEGs, shared between spatial and cellular analyses; LHX2 expression was stimulated by BMP7, inhibited by LDN193189 treatment and enriched in the pSMAD1/5-high MB tumor cells (Fig. 3B–E). pSMAD1/5 signal intensity and *LHX2* mRNA expression showed a significant positive correlation across all 18 ROIs in the two PDX tumors (Fig. 3F). Subsequently, we validated these findings in the four MB cell lines, and in two additional cell lines, DAOY (SHH MB) and D283 (Group 3/4 MB), and confirmed the induction of *LHX2* by exogenous BMP7 or BMP4 stimulation but not by TGFβ stimulation, the latter even exhibiting weak *LHX2* downregulation (Fig. 3G). Using the R2 platform (https://r2.amc.nl), we queried four independent cohorts of MB tumors [5, 33], and other types of malignant tumors (Supplementary Fig. S3). *LHX2* mRNA expression was significantly higher in MB, particularly in Groups 3, 4, and SHH, relative to normal human cerebellum (Supplementary Fig. S3A), or relative to several other tumors (Supplementary Fig. S3B). As a final validation of these findings, we relied on the patient TMA described above (Fig. 1D, E), which revealed significant co-expression patterns of pSMAD1/5 and LHX2 protein (Supplementary Fig. S4A). Quantification of the individual cells that scored positively for each of the pSMAD1/5 and LHX2, resulted in 7 out of 20 cores with moderate (*r* ≥ 0.4) or weaker (0.4 > *r* > 0.2) positive correlation (selected examples shown in Supplementary Fig. S4B). Furthermore, six out of six PDX models analyzed in Fig. 1A exhibited strong LHX2 protein expression in the pSMAD1/5-positive cells when compared to the corresponding pSMAD1/5-negative cells of the respective tumor (Supplementary Fig. S4C).

Next, we examined the signaling mechanism responsible for *LHX2* gene induction by BMP7 (Supplementary Fig. S5). Time-course experiments indicated that the onset of induction exceeded 6 hours of signaling and was detectable by 24 h (Supplementary Fig. S5A). The latter result might indicate indirect regulation of *LHX2* mRNA expression via secondary factors. In order to monitor the requirement of newly synthesized proteins as part of the *LHX2* gene response to BMP7, we inhibited protein synthesis via cycloheximide during the complete period of stimulation with BMP7 and monitored *LHX2* mRNA expression (Supplementary Fig. S5B). Although the cycloheximide treatment alone seemed to stabilize the *LHX2* mRNA by roughly twofold, there was no significant statistical difference compared to untreated samples. Nevertheless, upon BMP7 stimulation, the inducible mRNA levels were increased significantly to the same or even higher degree relative to control (Supplementary Fig. S5B). Simultaneous silencing of the three main SMAD mediators of BMP7 signaling, SMAD1, SMAD4, and SMAD5, in D425 MB cells, significantly inhibited BMP7-mediated induction of *LHX2* mRNA expression, to a similar extent as the inhibition of *ID1* induction (Supplementary Fig. S5C). Since many gene targets of BMP signaling require signaling inputs beyond SMADs, we analyzed the impact of protein kinase inhibitors against the c-Jun N-terminal kinase (JNK), the upstream activator of the MAP-kinases, MEK1 (MAP-kinase kinase/MAP2K1) and the p38 MAP-kinase (MAPK11), in D425 and MB002 cells, and compared their effects relative to the BMP receptor inhibitor LDN193189 (Supplementary Fig. S5D, E). Cell type-specific effects were recorded whereby the JNK inhibitor partially blocked *LHX2* induction by BMP7 in D425 cells and the MEK and p38 inhibitors partially blocked this response in MB002 cells (Supplementary Fig. S5D, E). These protein kinase inhibitors gave partial inhibition of the response, in contrast to complete inhibition by LDN193189, and weaker inhibitory effects compared to SMAD silencing (Supplementary Fig. S5; note that SMAD silencing efficiency was 35-50%). We then used the D425 cells in which the strongest inhibitory effect by the protein kinase inhibitors was recorded based on the JNK inhibitor (Supplementary Fig. S5D, E). We treated D425 cells transfected with the triple SMAD siRNA cocktail with the JNK inhibitor and observed that both basal and inducible *LHX2* mRNA levels were suppressed to a higher degree relative to the single perturbations (Supplementary Fig. S5F). We conclude that SMAD1/5/4 signaling with MAP-kinase (e.g., JNK) inputs mediate *LHX2* gene transcriptional induction in MB cells.

### LHX2 enhances BMP signaling

**Fig. 4:**
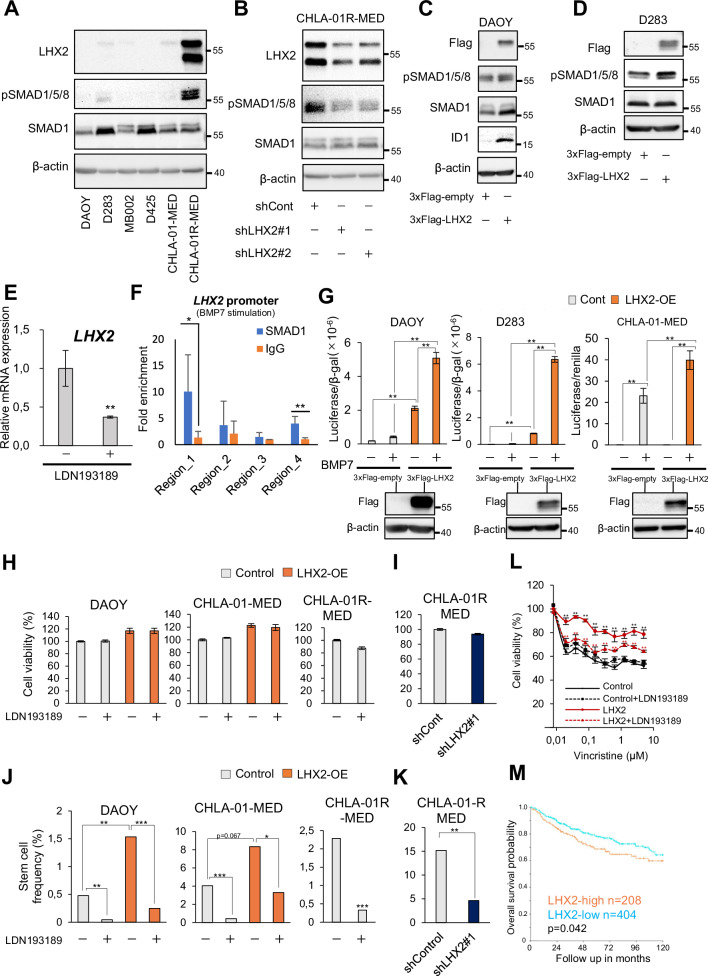
LHX2 enhances BMP signaling. Representative immunoblots of the indicated proteins in MB cell lines. Immunoblotting for Flag monitors transfected 3×Flag-LHX2 (**C**, **D**) and β-actin serves as loading control (**A**–**D**). The six MB cell lines analyzed in **(A)** were cultured under stem cell conditions. LHX2 knockdown clones of CHLA-01R-MED (shLHX2#1 and shLHX2#2) were analyzed in (**B**). DAOY cells stably expressing 3×Flag-LHX2 and the control clone were analyzed in (**C**). D283 cells with transient overexpression of 3×Flag-LHX2 were collected and analyzed 24 h after transfection in (**D**). Molecular mass markers (kDa) are shown. For original images, see Supplementary Fig. S12. **E** *LHX2* mRNA expression in DAOY cells stably expressing 3×Flag-LHX2 was measured by RT-qPCR 7 days after continuous culture with 0.2 µM LDN193189. The results were normalized to *GAPDH* levels. Data shown as the mean ± SD of three biological replicates. *p < 0.05, **p < 0.01 compared with control was assessed by two-tailed paired Student’s *t* test. **F** CUT&RUN assay in CHLA-01R-MED cells, treated with 100 ng/ml BMP7 or vehicle (-) for 3 days. Antibody against SMAD1 was used. qPCR data were normalized to the total amount of input chromatin and shown as fold-enrichment relative to IgG (negative control) for the BMP7-stimulated samples. Data shown as the mean ± SD were derived from three biological replicates. *p < 0.05, **p < 0.01, one-way ANOVA with Tukey test. Primer sequences are shown in Supplementary Table S5. **G** *BRE*_*2*_ luciferase reporter assays. DAOY, D283 or CHLA-01-MED cells with transient overexpression (OE, blue bars) of LHX2 or control (Cont) vector were stimulated with 100 ng/ml BMP7 or vehicle. Firefly luciferase was normalized to β-gal or renilla luciferase activity, as indicated. Mean values from 2 biological replicates, each with technical triplicates and corresponding SD are plotted. *p < 0.05, **p < 0.01, ***p < 0.001 compared to the indicated reference condition was assessed by one-way ANOVA with Tukey test. Below each diagram a corresponding immunoblot indicates the level of 3×Flag-LHX2 protein expression over β-actin loading control in the transfected cells and molecular mass markers (kDa) are shown. Proliferation assays in DAOY and CHLA-01-MED cells overexpressing (OE, blue bars) LHX2 or control vector (gray bars) (**H)** or in CHLA-01R-MED-shLXH2#1 cells (**I**). Cell proliferation was analyzed 72 h after treatment with 0.2 µM LDN193189 or DMSO (−) (**H**) or in the absence of any treatment (**I**) by PrestoBlue. Mean values from 3 biological replicates, each with technical triplicates and corresponding SD are plotted (**H**) or representative data shown as the mean ± SD of three technical replicates are plotted in (I). No significance was confirmed by one-way ANOVA with Tukey test. Average stem cell frequencies in DAOY and CHLA-01-MED cells overexpressing (OE, blue bars) LHX2 or control vector (gray bars) and in CHLA-01R-MED treated with 0.2 µM LDN193189 or DMSO (-) for 5 days (**J**) or in CHLA-01R-MED-shLHX2#1 compared to the control (**K**). Stem cell frequencies were calculated by ELDA and shown as bar graphs of mean values from 2 biological replicates, each with 8 technical replicates. Corresponding SD are not shown as the ELDA software generates only upper and lower limits of the measurements as shown in the original ELDA graphs of (**J**) and (**K**), in Supplementary Fig. S6C. *p < 0.05, **p < 0.01, ***p < 0.001 was assessed by chi-square test. **L** CHLA-01R-MED cells with or without LHX2 overexpression treated with the indicated concentration of vincristine, in combination or not with 0.2 µM LDN193189. Twenty-four hours after the treatment, cell viability was measured by PrestoBlue. Mean values from 3 biological replicates, each with technical triplicates and corresponding SD are plotted as percent viability relative to the control (100%). **M** Kaplan-Meier analysis demonstrating the association between *LHX2* mRNA expression and ten-year overall survival of 612 MB patients, plotted from R2: Genomics Analysis and Visualization Platform (https://r2.amc.nl). Data set: Cavalli et al. [5]. Based on the average *LHX2* mRNA expression, the 612 patients were divided into 2 groups (LHX2-high and -low). A *p*-value was calculated with a log-rank test.

Immunoblot analysis revealed significantly higher expression of LHX2 in CHLA-01R-MED cells, which also exhibited the highest levels of pSMAD1/5/8 relative to the other five cell lines (Fig. 4A). We consistently observed two LHX2 protein bands in cells that expressed high LHX2 levels (Fig. 4A), both of which were suppressed upon genetic silencing (Fig. 4B), thus possibly reflecting detection of the two major variants of LHX2 derived via alternative promoter use and/or splicing (Supplementary Methods). Note that the antibody used for immunoblot recognizes all three pSMAD1/5/8 proteins, whereas the antibody optimized for multispectral immunofluorescence analysis recognized only pSMAD1/5 (Supplementary Table S1). When LHX2 was stably silenced in CHLA-01R-MED cells by independent shRNAs, the autogenous pSMAD1/5/8 signals also decreased (Fig. 4B). Conversely, upon cloning the human *LHX2* cDNA from HeLa cells and overexpressing LHX2 in DAOY and D283 cells that express low endogenous LHX2 levels (Fig. 4A), pSMAD1/5/8 signals and ID1 expression were induced (Fig. 4C, D). It should be noted that the effect on pSMAD1/5/8 was stronger in D283 cells that have higher sensitivity to BMP/SMAD signaling compared to the DAOY cells that exhibit weaker sensitivity to BMP/SMAD signaling (Fig. 4A). This aspect of sensitivity of different MB cell models will be further illustrated below. *LHX2* mRNA was downregulated by the BMP receptor inhibitor LDN193189 (Fig. 4E; Supplementary Fig. S6A, B). The impact of LDN193189 was observed under 3D culture conditions of cells overexpressing LHX2 (Fig. 4E). Under these conditions, the MB cultures activate BMP ligand expression and signaling, a topic worth analyzing deeper in the future. Optimized chromatin immunoprecipitation using CUT&RUN assays with a SMAD1 antibody and primers detecting four distinct regions of the human *LHX2* promoter confirmed the recruitment of SMAD1 to two out of the four promoter sequences after BMP7 stimulation (Fig. 4F), suggesting direct regulation of *LHX2* transcription by BMP7-SMAD1 signaling. Moreover, LHX2 overexpression activated the BMP-responsive reporter *BRE*_*2*_-luc, derived from the *ID1* gene promoter [35], in all tested cell lines (Fig. 4G). However, this sensitive assay of BMP/SMAD signaling also demonstrated the weak signaling capacity of DAOY and D283 cells relative to the much stronger sensitivity of the CHLA-01-MED cells (Fig. 4G, empty vector conditions).

MB cell-based assays revealed no significant impact of inhibition of BMP receptor signaling or LHX2 silencing on proliferation (Fig. 4H, I), whereas significant effects on tumor-sphere formation were observed (Fig. 4J, K; Supplementary Fig. S6C). Quantification of the MB sphere assays clearly showed that LHX2 overexpression increased the frequency of stem-like cells in culture, and these effects were attenuated by inhibiting BMP signaling by LDN193189 (Fig. 4J, K; Supplementary Fig. S6C). These results suggest that BMP signaling and LHX2 high expression do not act as direct regulators of cell proliferation in vitro but rather have an impact on clonogenic (stem-like) capacity of the same cells in vitro. To investigate the clinical relevance of these findings, LHX2-overexpressing cells were treated with vincristine, a cytotoxic drug commonly used in MB treatment [36]. LHX2-overexpressing cells and BMP7-treated cells showed increased resistance to vincristine, which was attenuated when the treatment was combined with LDN193189 (Fig. 4L; Supplementary Fig. S6D). In agreement, patients expressing higher LHX2 levels exhibited poor prognosis compared to patients with lower LHX2 expression (Fig. 4M). Thus, gain-of-function of LHX2 in MB links to enhanced BMP signaling, generating a potential feed-forward loop.

### LHX2 induces ACVR1, forming a feed-forward BMP signaling loop

**Fig. 5:**
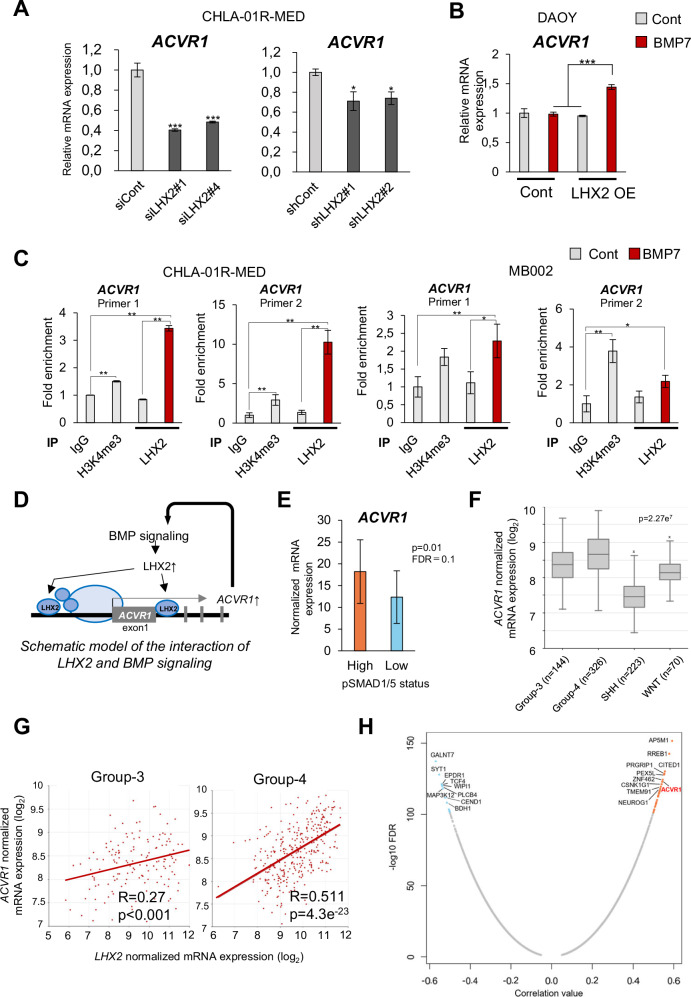
LHX2 induces *ACVR1* to form a feed-forward loop in BMP signaling. **A** *ACVR1* mRNA expression was analyzed in CHLA-01R-MED cells 48 h after transfection of siLHX2 (#1, #4, left panel), or from LHX2 stable knockdown clones (shLHX2#1, #2, right panel), by RT-qPCR. The results were normalized to *GAPDH* levels. Data shown as the mean ± SD are representative, each with three biological replicates. *p < 0.05, **p < 0.01 compared with control assessed by two-tailed paired Student’s *t* test. **B** *ACVR1* mRNA expression analyzed by RT-qPCR in DAOY cells stably overexpressing (OE) LHX2. Cells were stimulated with vehicle (-) or 100 ng/ml BMP7 for 3 days. Data shown are representative, each with three technical replicates. *p < 0.05, **p < 0.01 by one-way ANOVA with Tukey test. **C** CUT&RUN assay in CHLA-01R-MED and MB002 cells, treated with 100 ng/ml BMP7 or vehicle (-) for 3 days. Antibodies against LHX2 and H3K4me^3^ were used. qPCR data were normalized to the total amount of input chromatin and shown as fold-enrichment relative to IgG (negative control). Data shown as the mean ± SD are representative, each with three technical replicates. *p < 0.05, **p < 0.01, one-way ANOVA with Tukey test. Primer sequences are shown in Supplementary Table S5. **D** Schematic model of the interaction of LHX2 with two regulatory sequences on the *ACVR1* gene resulting in RNA polymerase II (light-colored oval) and cofactors (small ovals) mediating transcription and consequent enhanced BMP signaling. **E** *ACVR1* mRNA expression in 18 regions of interest (ROIs) analyzed by NanoString GeoMx^®^ profiling representing the two Group 4 PDXs, stratified as pSMAD1/5-high and -low tumor cells. FDR represents adjusted *p*-value. **F**, **G** mRNA expression in MB was plotted from R2: Genomics Analysis and Visualization Platform (https://r2.amc.nl). Data set: Cavalli et al. [5]. *ACVR1* expression in each subgroup of MB is shown as a box plot with horizontal lines indicating the median, whiskers indicating the SEM and numbers (*n*) of samples indicated (**F**). Correlation plots show a positive correlation between *ACVR1* and *LHX2* mRNA expression in Group 3 (left) and Group 4 (right) MB (**G**). Spearman correlation values (*R*) and associated *p*-values derived by the Wilcoxon rank sum test are shown. **H** Correlation analysis of *LHX2* mRNA expression relative to the expression of all protein-coding genes across 1641 MB sample plotted from R2: Genomics Analysis and Visualization Platform (https://r2.amc.nl). Data set: Weishaupt et al. [46]. Gene expression in log10 FDR is plotted against the correlation (*r*) value showing only significantly regulated genes. Individual gene names are indicated, with those being the most highly correlated and higher expressed shown in red and those anti-correlated in blue.

We investigated the underlying mechanism by which LHX2 enhances BMP signaling. Transient and stable LHX2 knockdown with distinct si/shRNAs in CHLA-01R-MED showed downregulation of *ACVR1* mRNA levels (Fig. 5A), whereas LHX2 overexpressing DAOY clones showed increased *ACVR1* expression after stimulation with BMP7 (Fig. 5B). Furthermore, CUT&RUN assays revealed LHX2 binding to two different regions, one upstream and another downstream of the transcriptional start site (TSS) of the *ACVR1* gene (Fig. 5C, D). Both genomic *ACVR1* regions were positive for the histone-3 lysine-4 trimethylation (H3K4me^3^) mark characterizing transcriptionally active proximal promoters (Fig. 5C). Interestingly, the spatial tumor analysis revealed relatively higher expression levels of *ACVR1* in pSMAD1/5-high ROIs (*p*-value = 0.01, FDR = 0.1) (Fig. 5E). The large MB cohort transcriptomic data queried from the R2 platform also revealed higher mRNA expression of *ACVR1* in Groups 3 and 4 compared to the rest of the MB groups (Fig. 5F). A significant positive correlation between *LHX2* and *ACVR1* mRNA expression was observed particularly in Group 4 tumors (Fig. 5G). Additional analysis between *LHX2* mRNA expression and all protein-coding genes across 1 641 MB samples identified *ACVR1* (*r*-value = 0.54, FDR = 1.01e^−123^) among the top 10 positively-correlated genes (Fig. 5H). These data on LHX2 and BMP signaling co-regulation align well with the cellular RNA-seq data, where a robust induction of *ACVR1* expression by stimulation with BMP7 in CHLA-01R-MED, D425, and MB002 cells could be observed (Supplementary Fig. S7, S8A). Examining carefully mRNA expression patterns of all BMP receptors and ligands indicated stronger upregulation of BMP receptors compared to BMP ligands (Supplementary Fig. S7). In addition to *ACVR1*, *BMPR1A* and *BMP7* expression levels were regulated by BMP signaling in multiple cell models (Supplementary Fig. S8A). Analyzing the four *SMAD* mRNAs of the BMP signaling pathways showed only minor effects in the same four cell models, with marginal positive or negative regulation in single-cell lines, possibly suggesting a lack of biological significance (Supplementary Fig. S8A). CUT&RUN analysis of LHX2 association with proximal gene promoters under similar conditions as the assays performed for *ACVR1* (Fig. 5C) failed to demonstrate specific enrichment of LHX2 to sequences of the other five BMP receptors genes (Supplementary Fig. S8B). These data agree with genome-wide searches that identified the LHX2 binding motif primarily in intergenic and intronic regions, with only 1% of their total number mapping within promoter regions (Supplementary Fig. S9A, B). Gene ontology analysis of the genes harboring LHX2 binding motifs within 1 kbp from their TSS identified primarily genes involved in brain functions, including sensory perception, and only a few genes involved in signal transduction (Supplementary Fig. S9C). Among the latter is *ACVR1*, which apparently represents a gene selected during evolution to participate in the brain-specific LHX2 regulatory network. Examination of genetic mutations in *ACVR1* identified among 4586 pediatric brain tumor cases, only two missense mutations mapping one in the extracellular domain and the other in the C-terminal part of the protein kinase domain of the receptor (Supplementary Fig. S9D), both exhibiting as of today the absence of functional relevance, while the published record has not yet reported *ACVR1* mutations in MB genomes.

We finally analyzed gain-of-function of ACVR1 over LHX2 loss-of-function in MB002 cells that endogenously express LHX2 (Supplementary Fig. S10). Upon stable silencing of LHX2 (Supplementary Fig. S10A) and transient infection with adenoviral vectors expressing control LacZ or constitutively active ACVR1 receptor, the endogenous *ID1* and *LHX2* mRNA levels were induced (Supplementary Fig. S10B), indicating that gain-of-function of ACVR1 can revert the phenotype of LHX2 loss-of-function, despite the persistent silencing of LHX2, which allows only weak reversion of LHX2 levels. Under these conditions, the sphere-forming capacity of the LHX2 silenced cells was increased after constitutively active ACVR1 expression (Supplementary Fig. S10C), indicating the rescuing ability of the active receptor over a low LHX2 background. Thus, LHX2 acts transcriptionally to maintain the high responsiveness of MB cells to BMP signaling by regulating ACVR1 expression. Transcriptional induction of *ACVR1* expression, rather activating mutations, appears to be relevant for BMP signaling activation in MB.

### LHX2 promotes the oncogenic activity of BMP signaling

**Fig. 6:**
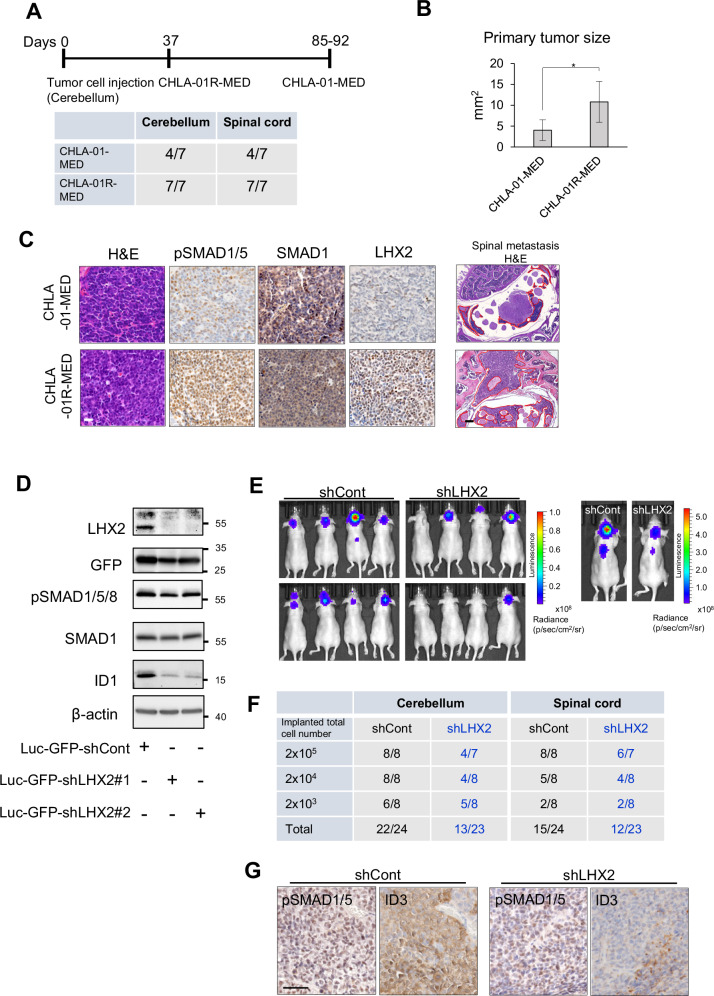
LHX2 positively contributes to MB tumor propagation. **A** Experimental timeline of the in vivo study (top). CHLA-01-MED or CHLA-01R-MED cells were orthotopically transplanted into the cerebellum of mice (*n* = 7 per cell line). The mice were sacrificed when body weight loss was higher than 10% of the original weight. The dates on which the mice were sacrificed are shown. The table shows the number of primary tumors and spinal metastases confirmed by histological assessment (bottom). **B** Primary tumor size was measured on hematoxylin and eosin (H&E) stained slides by QuPath. The bar graph shows mean values and SD derived from the four or seven independent data points. **C** H&E staining and immunohistochemistry for pSMAD1/5, SMAD1, and LHX2 of the orthotopic xenograft of CHLA-01-MED and CHLA-01R-MED cells. Primary tumors in the cerebellum (left, scale bar 20 µm) and spinal metastases (right, scale bar 200 µm). **D** Representative immunoblots of CHLA-01R-MED luc-GFP-shLHX2#1, #2, and Luc-GFP-shControl cells analyzed prior to orthotopic transplantation. LHX2, GFP (internal control expressed by the lentiviral shRNA vectors), C-terminally phosphorylated SMAD1/5/8, SMAD1, ID1, and β-actin (loading control) are shown along with molecular mass markers (kDa). For original images, see Supplementary Fig. S12. **E** In vivo bioluminescence imaging of orthotopic xenografts (in 8 mice in total) generated by 2 × 10^4^ CHLA-01R-MED cells carrying control (shCont) or LHX2-specific (shLHX2) shRNAs, and imaged at 2.5 weeks post-transplantation (left). The images on the right show single mice in which 2 × 10^5^ cells were orthotopically transplanted in order to visualize both primary tumors and spinal metastases. The luminescence scale is shown in photons (p) per sec per cm^2^ per steradian (sr). **F** Table listing the number of primary tumors and spinal metastases confirmed by bioluminescence imaging for the three different cell populations transplanted per cerebellum. **G** Immunohistochemistry for pSMAD1/5 and ID3 of representative primary tumors after orthotopic xenografts of CHLA-01R-MED cells carrying shCont or LHX2-shRNA (scale bar 50 µm).

We orthotopically transplanted nude mice to analyze the potential effect of LHX2 on primary tumor growth. We transplanted the two isogenic Group 4 cell lines, CHLA-01-MED and CHLA-01R-MED, known to generate tumors [34], since the PDX models were not practically amenable due to their very long time of growth. Notably, CHLA-01R-MED cells, which express significantly high levels of LHX2 and pSMAD1/5/8 in culture (Fig. 4A), showed more efficient and rapid tumor formation (Fig. 6A) and gave rise to larger tumors compared to CHLA-01-MED (Fig. 6B). Histological analysis confirmed the higher pSMAD1/5 and LHX2 levels in tumors generated by CHLA-01R-MED cells relative to the tumors originating from CHLA-01-MED cells, and the former exhibited a more invasive phenotype leading to spinal metastasis (Fig. 6C). To causally link LHX2 to primary tumor growth, we transplanted CHLA-01R-MED cells engineered to stably silence LHX2 using shRNAs (Fig. 6D) and expressing luciferase, compatible with bioluminescence imaging (Fig. 6E-G). Compared to control cells, *LHX2* stable silencing did not impair tumor growth in vivo (Supplementary Fig. S11), an observation similar to the in vitro data where *LHX2* knockdown failed to impact cell viability (Fig. 4H, I). We then evaluated the potential impact of *LHX2* manipulation on tumor propagation efficiency by performing an in vivo ELDA utilizing serially diluted cells transplanted orthotopically (Fig. 6F). Silencing of LHX2 in the engrafted tumor cells led to a distinct reduction in primary tumor formation (Fig. 6E, Supplementary Fig. S11B). Furthermore, signals detected as spinal metastasis by bioluminescence imaging were also significantly reduced after silencing of LHX2; however, histologic examination of the micrometastases upon dissection of the spinal cords could support only an inhibitory trend on micrometastases caused by the reduction of LHX2 (Fig. 6F). As established in the PDX models (Fig. 1) and in the transplantation experiments (Fig. 6C), pSMAD1/5 and ID3, both marking endogenous BMP signaling in the tumor cells, were reduced in MB tumors depleted of LHX2 (Fig. 6G). We also analyzed two more MB tumor models after orthotopic transplantation of MB002 and D425 cells; both MB cell lines generated tumors that scored double-positively for pSMAD1/5 and LHX2 (Supplementary Fig. S11C), although nuclear LHX2 staining was weaker. Counterstaining for total SMAD1 in these tumors indicated that the intensity of pSMAD1/5 signals did not reflect the strong expression levels of SMAD1 (Supplementary Fig. S11C), suggesting that the efficiency of signaling must depend on BMP ligand and receptor levels in the tumors. It is worth highlighting that although these two MB cell models express weakly detectable pSMAD1/5 and LHX2 protein levels when cultured in suspension, forming spheroids (Fig. 4A), the corresponding MB tumors exhibited detectable pSMAD1/5 and LHX2 protein levels (Supplementary Fig. S11C). These experiments, in combination with the initial analysis of the PDX MB models (Fig. 1), highlight a BMP signaling-LHX2 functional unit in MB growth and survival in vivo.

## Discussion

BMP signaling can favor pro-oncogenic or anti-tumorigenic fate in different MB cell cultures, a fact that correlates with BMP actions during normal mouse cerebellar development, and probably underscores the specificity of biological responses of the cell-of-origin of each particular MB tumor cell to BMP signaling [3]. Thus, by undertaking a systematic analysis of BMP signaling in MB (6 MB cell lines, 7 PDX MB models, and complementary human TMA analysis), we report that MB Groups SHH, 3 and 4 exhibited detectable and strongly pSMAD1/5-positive cell populations, which indicated active BMP signaling in MB tumors in vivo. Note that the WNT MB Group was not represented in our analyses due to the scarcity of WNT MB models. Overall, while our study covered a rather extensive ground, since the Group 4 MB PDX models were not practically amenable due to the very long time of the tumor latency following transplantation, the acquired data do not provide discriminatory power in order to assign preferential action of BMP signaling in a single or specific group of MB patients. Utilizing comparative transcriptomic analyses of Group 4 tumors and SHH, Group 3 and Group 4 MB cell models responding to BMP7, we identified the homeobox transcription factor LHX2 operating downstream of, and enhancing, BMP signaling.

MB patient data support the high expression of BMP ligands (especially BMP7), ID3, and ID4, essentially in all MB groups, abundant levels of pSMAD1/5 in patient-derived (PDX Group 3, Group 4 and SHH) tumor samples, and robust signaling activation by BMPs in MB cell cultures. These data suggest that all forms of MB may be under the influence of BMP signaling during a period of their history, however, selective enrichment of BMP signaling in specific MB subgroups remains possible (Fig. 1). Furthermore, we provide evidence for a positive role of BMP signaling (in association with LHX2, see below) in MB 3D tumor-sphere growth, survival and resistance to vincristine. This finding supports evidence from diverse tumor types for the role of TGF-β/BMP family members providing resistance to treatment [37]. Since the response of MB cells to BMP signaling can be either pro-oncogenic or growth suppressive [3], our evidence favors the existence of subgroups of MB patients in which BMPs act in an oncogenic manner, at least in part, via the action of the transcription factor LHX2.

LHX2 has an established function in brain development, as it regulates Notch pathway components in differentiating neuronal cells [38]. Guidance of neuronal axons is driven by LHX2, which regulates expression of the guidance proteins Robo1 and Robo2 in the mouse thalamic cortex [39]. Mechanistically, LHX2 controls chromatin accessibility, acting as a pioneering factor, thus facilitating binding of cooperating transcription factors, as demonstrated in retinal progenitor cells [40]. In MB, LHX2, together with the transcription factors HLX, EOMES, and LMX1A, has been proposed, but not shown directly, to mark progenitor cells that mutate and develop to MB [17]. However, to the best of our knowledge, no specific functional and mechanistic attribute of LHX2 has yet been investigated in pediatric MB. Interestingly, our transcriptomic and bioinformatic analyses of PDX MB revealed a significant down-modulation of crucial pathways, including SLIT/ROBO signaling in pSMAD1/5-high, i.e., LHX2-high, MB cells (Fig. 2). This agrees with the reported mechanism whereby Lhx2 downregulates Slit/Robo signaling in mouse thalamocortical neuron development [39]. Thus, our data resource opens ground for analyzing the role(s) LHX2 may play in MB.

*LHX2* mRNA expression correlated with BMP signaling activity defined by pSMAD1/5 staining intensity in the tested MB PDXs. LHX2 was induced by exogenous stimulation with BMPs in diverse MB cell cultures, via SMAD and MAP-kinase signaling, exhibiting relatively slow kinetics of induction (over 6 h of signaling) and independence from newly synthesized proteins. LHX2 expression peaked in MB cells exhibiting the highest BMP signaling activity, whereas, LHX2 depletion negatively regulated autogenous BMP signaling. Similar to BMP signaling, whose inhibition suppressed tumor-sphere growth, LHX2 positively contributed to 3D tumor-sphere formation, and inhibition of BMP signaling suppressed the LHX2 effects on tumor-sphere propagation. Furthermore, LHX2 overexpression promoted chemoresistance in MB cultures, and LHX2-high MB patients showed poor clinical outcome. These data propose that MB tumors are under the influence of cooperative activities of LHX2 and BMP signaling. Mechanistically, we found that LHX2 binds to two distinct regulatory sequences of *ACVR1*, one of the BMP type I receptors, thus forming a feed-forward loop that further enhances BMP signaling. Efforts to query similar transcriptional regulation of all other BMP family receptors were not successful. So far, evidence for mutational activation of ACVR1 or other BMP receptors in MB does not exist, despite the existence of specific single nucleotide polymorphisms in the *ACVR1* gene. This appears different from evidence on *ACVR1* mutations that constitutively activate BMP signaling in diffuse intrinsic pontine glioma [41–43]. However, it should be appreciated that the number of pontine glioma patients with activating *ACVR1* mutations is limited, and BMP signaling can take both tumor suppressive and pro-tumorigenic actions [43], thus highlighting the need of deeper molecular studies on the roles that BMP signaling plays in brain tumors in general.

The mechanism of LHX2-mediated regulation of BMP signaling via ACVR1 established here in MB cells has not, to the best of our knowledge, been previously reported. Processes governing optical epithelial differentiation during early mouse eye organogenesis involve Lhx2, whose function is required for *Bmp4* and *Bmp7* expression, via, as yet, uncharacterized molecular mechanism [44]. Furthermore, similar to our findings in MB, analysis of the impact of BMP6 and BMP7 on dorsal interneuron explants from the developing rat spinal cord, reported a positive correlation between BMP7 signaling and the generation of Lhx2/Lhx9 double-positive cells [45], suggesting that regulation of LHX2 by BMP signaling may be relevant to multiple neuronal cell types, independent of malignant development.

Our animal experiments also support a positive role of LHX2 and BMP signaling in MB tumor propagation. Although the impact of LHX2 depletion was statistically significant, it did not exhibit an all-or-nothing pattern, which is compatible with the requirement of cooperating transcription factors in the establishment of MB [6, 7, 9, 17]. We also attempted to block ACVR1 kinase activity using chemical inhibitors in order to formally associate ACVR1 activity downstream of LHX2. However, all such experiments led to the lethality of the transplanted animals, consistent with the established toxicity and side effects of such inhibitors. Future genetic perturbation of the *ACVR1* gene is worth examining to address this important point. It is also worth noting that the increased ability for tumor initiation exhibited by MB cells expressing higher LHX2 levels, correlated well with metastatic potential but did not correlate with the impact of LHX2 on cell viability/proliferation. This suggests that the cancer stem cell frequency in vitro and in vivo is probably more complicated than a simple effect of LHX2 on cell viability and proliferation. Rather, this effect of LHX2 may have to do with the ability of the tumor cells to sustain tumorigenic potential over time, as exemplified by cell colonies grown in vitro and tumor maintenance in the transplanted animals.

BMP stimulation induced LHX2 expression in all tested MB cultures, including SHH, Group 3 and Group 4 MB. Unfortunately, suitable models of WNT MB are sparse, likely because it is the least common MB subgroup with undoubtedly the best prognosis [2]. As is the case for most tumors, future analysis should aim to include larger cohorts of patient tissue and primary cell cultures in order to identify subpopulations of MB patients in which BMPs and LHX2 provide oncogenic potential and those where they have minimal impact. Such analysis may validate BMP signaling as a predictive and/or diagnostic marker of tumor development in relevant MB patients.

In conclusion, we have demonstrated that BMP signaling induces the transcription factor LHX2, which enhances the oncogenic activities of BMP signaling in MB. Our study opens new ground for the understanding of BMP signaling in MB and provides useful insights into the role of the ACVR1 receptor in MB patients.

## Supplementary information

Supplementary File

Supplementary Table S2

Supplementary Table S3

Supplementary Table S4

## Data Availability

Source data are provided in this paper. RNA sequencing data are deposited to the public repository GEO (accession number GSE229150).
