# Supplementary material for: The transcription factor LHX2 mediates and enhances oncogenic BMP signaling in medulloblastoma
Source: Cell Death Differ. 2025 Mar 28;32(10):1915–29. doi: 10.1038/s41418-025-01488-6 (PMC12501261; doi:10.1038/s41418-025-01488-6)
Supplement: Supplementary file 1 — Supplementary File [file 41418_2025_1488_MOESM1_ESM.pdf]

The transcription factor LHX2 mediates and enhances oncogenic BMP signaling in medulloblastoma

Yae Ohata[1], Mohamad M. Ali[1], Yutaro Tsubakihara[1], Yuka Itoh[1,2], Gabriela Rosén[3], Tobias Bergström[3], Anita Morén[1], Irene Golán-Cancela[1], Ayana Nakada[1,4], Oleksandr Voytyuk[1], Maiko Tsuchiya[5,6], Rei Fukui[7], Kouhei Yamamoto[8,9], Paula Martín-Rubio[10], Patricia Sancho[10], Carina Strell[3,11], Patrick Micke[3], Robert J. Wechsler-Reya[12], Yoshinobu Hashizume[13], Kohei Miyazono[14], Laia Caja[1], Carl-Henrik Heldin[1], Fredrik J. Swartling[3], and Aristidis Moustakas[1]

[1]Department of Medical Biochemistry and Microbiology, Science for Life Laboratory, Box 582, Biomedical Center, Uppsala University, SE-751 23 Uppsala, Sweden.
[2]Department of Biochemistry, Graduate School of Medicine, University of Yamanashi, 1110 Shimokato, Chuo, Yamanashi 409-3898, Japan.
[3]Department of Immunology, Genetics and Pathology, Rudbeck Laboratory, Science for Life Laboratory, Uppsala University, SE-751 85 Uppsala, Sweden.
[4]Faculty of Pharmaceutical Science, The University of Tokyo, Hongo 7-3-1 Bunkyo-ku, Tokyo, 113-8655 Japan.
[5]Department of Oral Pathology, Tokyo Medical and Dental University, Yushima 1-5-45 Bunkyo-ku, Tokyo, 113-8510, Japan.
[6]Department of Pathology, Teikyo University School of Medicine, Kaga 2-11-1 Itabashi-ku, Tokyo, 173-8605, Japan.
[7]Department of Pathology, Nihon University School of Dentistry, 1-8-13 Kanda-Surugadai, Chiyoda-ku, Tokyo 101-8310, Japan.
[8]Department of Comprehensive Pathology, Graduate School of Medical and Dental Sciences, Tokyo Medical and Dental University, Tokyo 113-8510, Japan.
[9]Department of Human Pathology, Graduate School of Medical and Dental Sciences, Tokyo Medical and Dental University, Tokyo 113-8510, Japan.
[10]Translational Research Unit, Hospital Universitario Miguel Servet, IIS Aragon, Zaragoza, Spain.
[11]Center for Cancer Biomarkers (CCBIO), Department of Clinical Medicine, University of Bergen, Bergen, Norway.
[12]Herbert Irving Comprehensive Cancer Center, Columbia University Medical Center, 1130 St. Nicholas Ave., ICRC 10-1004, New York, NY 10032, USA.
[13]RIKEN Program for Drug Discovery and Medical Technology Platforms, 2–1 Hirosawa, Wako, Saitama, 351–0198 Japan.
[14]Department of Applied Pathology, Graduate School of Medicine, The University of Tokyo, Hongo 7-3-1 Bunkyo-ku, Tokyo, 113-0033 Japan.

Running title: Oncogenic actions of LHX2 and BMP in medulloblastoma

Supplementary information

Supplementary data for this article are available online: Methods, references, 5 supplementary tables and 12 supplementary figures.

Supplementary Materials and Methods

Reagents and treatments

BMP7 (100 ng/ml, a kind gift from K. Sampath, Sanofi-Genzyme Cambridge Research Center, Cambridge, MA, USA) and BMP4 (100 ng/ml, PeproTech EC Ltd/Thermo Fisher Scientific, Stockholm, Sweden) were used as indicated in the figure legends. The protein synthesis inhibitor cycloheximide (40 µg/ml) dissolved in PBS was applied for 48 h simultaneously with the incubation with BMP7, as indicated in the figure legends. The BMP type I receptor inhibitor LDN193189 (synthesized at RIKEN) was used as indicated in the figure legends. The c-Jun N-terminal kinase (JNK) inhibitor SP600125 (Merck/Calbiochem, Stockholm, Sweden), the MAP-kinase kinase 1 (MEK) inhibitor PD184352 (Sigma-Aldrich AB, Stockholm, Sweden) and the p38 MAP-kinase inhibitor SB203580 (Merck/Calbiochem, Stockholm, Sweden) were used as indicated in the figures. All chemical inhibitors were dissolved in dimethyl-sulphoxide (DMSO), which was also used as vehicle in control treatments.

Human MB cell lines

MB cell lines DAOY (SHH), D425 and MB002 (Group 3), D283 (Group 3/4) were described [1, 2], CHLA-01-MED (Group 4, CRL-3021) and CHLA-01R-MED (Group 4, CRL-3034) were purchased from the American Type Culture Collection (https://www.atcc.org/). DAOY, D425 and D283 were cultured in Dulbecco's modified

Eagle's medium (DMEM, Sigma-Aldrich AB, Stockholm, Sweden), supplemented with 10% fetal bovine serum (FBS; Biowest, Almeco A/S, Esbjerg, Denmark) and 100 U/ml penicillin, 100 µg/ml streptomycin (Sigma-Aldrich AB, Stockholm, Sweden). MB002 were maintained in 1:1 DMEM/F12 Glutamax-Neurobasal medium (Thermo Fisher Scientific/Life Technologies, Stockholm, Sweden), supplemented with B27, non-essential amino acids, 1 mM Na-pyruvate, 25 mM Hepes, 20 ng/ml epidermal growth factor (EGF), 20 ng/ml basic fibroblast growth factor (bFGF), 10 ng/ml leukemia inhibitory factor, 2 ng/ml heparin, 100 U/ml penicillin and 100 µg/ml streptomycin. CHLA-01-MED and CHLA-01R-MED were maintained in DMEM/F12 supplemented with B27, 20 ng/ml EGF, 20 ng/ml bFGF, 100 U/ml penicillin and 100 µg/ml streptomycin. D283 and DAOY were cultured as 3D-neurospheres in DMEM supplemented with B27, 20 ng/ml EGF, 20 ng/ml bFGF, 100 U/ml penicillin and 100 µg/ml streptomycin. All cell lines were cultured at 37 ^{o}C under a 5% CO_2 atmosphere, were free of mycoplasma (tested every 4 months) and were authenticated using PCR-single-locus-technology (Eurofins, Uppsala, Sweden).

Lentiviral, adenoviral vectors and infections

The lentiviral vectors encoding short hairpin (sh) LHX2 and a green fluorescent protein (GFP) (GFP-shLHX2#1, GFP-shLHX2#2) were generated by Gateway technology (Thermo Fisher Scientific/Invitrogen, Stockholm, Sweden). Two shRNA sequences against distinct *LHX2* mRNA regions (shLHX2#1, 5´-GCGCTAAGCTGCAACGAAA-3´; shLHX2#2, 5´-GCGCTCGGGACTTGGTTTA-3´) and GFP-shControl, non-targeting sequence (5´- ATCTCGCTTGGGCGAGAGTAAG-3´), were used. CHLA-01R-MED and MB002 cells were transduced with the lentiviral particles and pools of infected cells were used. For the generation of luciferase-expressing clones (luc-GFP-shCont, luc-

GFP-shLHX2#1, luc-GFP-shLHX2#2), CHLA-01R-MED cells were first transduced with lentivirus delivering luciferase (pLenti-*CMV*-puro-luc) and selected by 1 μg/ml puromycin for 48 h. Subsequently, luciferase-expressing cells were infected with shRNA particles as described above.

Constitutively active ad-caALK2/ACVR1 or control ad-LacZ viruses, were amplified, titrated and used as previously described by us [3]. MB002 and CHLA-01R-MED cells were transiently infected with these viruses with multiplicity of infection 5 (moi 5) for 24 h in 12-well plates, centrifuged and washed with fresh complete medium before transfer to 6-well plates, where they were cultured for another 24 h, then counted and 2×10^5 cells were plated into low attachment 96-well plates for the extreme limiting dilution assay or in 12-well plates for mRNA and protein analysis as described below.

siRNA or plasmid transfections

Transient transfections with siRNAs (final concentration 20 nM) or plasmids were performed using *Tras*IT-X2 reagent (Mirus Bio LLC, Madison, WI, USA), following the manufacturer's instructions. When siRNA transfections were combined with chemical inhibitor treatments, the transfected cells were cultured according to the manufacturer's instructions, and chemical inhibitor or solvent DMSO were added during the last 24 h of the culture. ON-TARGETplus (Horizon Discovery/Dharmacon, Cambridge, UK) human *LHX2* siRNA set of 4 LQ-019197-01-0005, human *SMAD1* siRNA set of 4 LQ-012723-01-0005, human *SMAD4* siRNA set of 4 LQ-040687-01-0005, human *SMAD5* siRNA set of 4 LQ-015791-01-0005, and Non-targeting Pool D-001810-10-20 were used. Human *LHX2* cDNA generated from cervical carcinoma

HeLa cells was cloned into pcDNA3-3×Flag through *HIND III*/*EcoRI* and sequencing verified identity to the human reference sequence. DAOY cells were stably transfected with pcDNA3-3×Flag-LHX2 for 48 h before selection with 1 mg/ml geneticin (Thermo Fisher Scientific, Stockholm, Sweden) for two weeks. D283 and CHLA-01-MED cells were transiently transfected with pcDNA3-3×Flag-LHX2 and/or reporter plasmid pBRE_2-luc [4] for 48 h.

Mouse transplantation experiments and bioluminescence imaging

A total of 2×10^5 CHLA-01-MED or CHLA-01R-MED cells were transplanted into athymic female Nude-Foxn1nu mice by stereotactic intracranial transplantation, as described [5]. Serial dilutions (2×10^5, 2×10^4, 2×10^3) of CHLA-01R-MED-luc-shCont and -luc-shLHX2#1 cells were transplanted and bioluminescece was imaged once or twice per week. The mice were sacrificed when more than 10% of weight loss was observed. Brains and spinal cords were dissected and processed for histological assessment. Primary tumor area was measured based on hematoxylin & eosin staining using QuPath, https://qupath.github.io/ [6]. Mouse husbandry and manipulations followed the national guidelines after approval by the Uppsala University Animal Experiment Ethics Board.

For MB002 and D425, a total of 5×10^4 cells were transplanted into 4-week old athymic Nude-Foxn1nu mice by stereotactic intracranial transplantation into the cerebellum under isofluorane-induced anesthesia. Eight mice per condition were included in every in vivo assay and this number was calculated as sufficient in order to provide us with power for statistical analysis of differences between conditions that were compared (shControl to shLHX2, and serial dilution analyses). Mice were

selected for each group of eight, without a specific randomization protocol as all mice were genetically identical, produced as a single litter. The mice were sacrificed when more than 10% of weight loss or changes in mobility were observed, after approximately 4 to 8 weeks. Tumors were collected and weighted and processed for histological assessment. Mice were housed according to institutional guidelines and all experimental procedures were performed in compliance with the guidelines for the welfare of experimental animals as approved by the Universidad of Zaragoza Ethics Committee (CEICA PI34/21).

Six minutes after intrapreneurial administration of 75 mg/kg sodium-D-luciferin (LUCNA; Gold Biotechnology Inc., Olivette, MO, USA) in saline, *in vivo* bioluminescence imaging was performed in an IVIS-Spectrum instrument with the corresponding software (PerkinElmer Sverige AB, Upplands Väsby, Sweden). Contours were drawn around cranial and spinal signals and mean values of photons/(sec.cm^2.steradian) were recorded.

All the procedures were performed in accordance with the guidelines for Ethical Conduct in the Care and Use of Animals as stated in The International Guiding Principles for Biomedical Research involving Animals, developed by the Council for International Organizations of Medical Sciences (CIOMS).

Luciferase assays

DAOY, D283 and CHLA-01-MED cells were transiently transfected with the BMP/SMAD responsive promoter reporter construct (p*BRE*$_2$-luc) and p*CMV*-β-gal (encoding β-galactosidase) or *TK-Renilla* luciferase reporter plasmid (p*GL4.74*, Promega, Madison, WI, USA) for normalization of luciferase activity, quantified using

the Firefly and Renilla Dual Luciferase Assay kit (BTIU30003-2, Biotium, Fremont, CA, USA). Relative normalized luciferase activity expressed as mean values derived from triplicate determinations with standard deviations, is presented graphically.

Proliferation and cell viability assays

DAOY or CHLA-01-MED cells transiently transfected with pcDNA3-3×Flag-LHX2 or parental CHLA-01R-MED cells and stable shLHX2#1 clones were incubated with either DMSO (control) or 0.2 µM LDN193189 for 3 days. Alternatively, CHLA-01-MED cells transiently transfected with control vector or pcDNA3-3×Flag-LHX2 were treated with vincristine in serial dilutions (as explained in the figure legend) for 24 h in the presence or absence of 0.2 µM LDN193189. CHLA-01-MED cells pretreated with 100 ng/ml BMP7 for 7 days were incubated with vincristine. Cell proliferation was analyzed by PrestoBlue reagent (Thermo Fisher Scientific, Stockholm, Sweden), following the manufacturer's protocol.

Extreme limiting dilution assay (ELDA)

DAOY, CHLA01-MED, CHLA-01R-MED, MB002 cells and MB002 cells after adenoviral infections were seeded on low-attachment 96-well plates in serially decreasing numbers (6 replicates per condition) and spheres were analyzed using the ELDA program (http://bioinf.wehi.edu.au/software/elda).

Multispectral immunofluorescence and immunohistochemistry

Formalin-fixed, paraffin-embedded (FFPE) samples of PDX MB (Group 4 DMB006, RCMB029, SHH RCM018, BT-084, Icb-984MB, and Group 3 Med-511-FH, Med-211-

FH), orthotopic xenografts of CHLA-01-MED and CHLA-01R-MED and a commercially available human MB tissue microarray (TMA, carrying 20 human MB cores, Biomax, US, BC17012c, from TissueArray.Com LLC, Derwood, MD, USA) were analyzed. The TMA did not include any specific annotation of the tumors. Histological and immunohistochemical evaluation by us identified 10 SHH (characterized by desmoplastic/nodular morphology and/or expression of YAP1 and no nuclear immunoreactivity for β-catenin) and 10 non-WNT/non-SHH (negative for YAP1 and for nuclear β-catenin) MB tumors. We therefore annotated the TMA as including 10 SHH and 10 non-WNT/SHH MB tumor cores. For antigen retrieval, slides were boiled in pH 6.0 or pH 9.0 buffer for 15 min, using a microwave oven, and incubated with primary antibodies (Supplementary Table S1) for 30 min at room temperature or overnight at 4°C. Anti-rabbit/mouse Opal Polymer-horseradish peroxidase ready-to-use immunohistochemistry detection reagents (ARH1001EA, PerkinElmer Sverige AB, Upplands Väsby, Sweden) were used as secondary antibodies. For staining of PDX tumors, slides were incubated with Opal fluorophores (570 nm) for 10 min at room temperature, followed by DAPI (4',6-diamidino-2-phenylindole) staining and mounting with ProlongTM Diamond Antifade mountant (Thermo Fisher Scientific, Stockholm, Sweden). Image analysis using the Vectra-Polaris (PerkinElmer Sverige AB, Upplands Väsby, Sweden) inForm software applied spectral unmixing, cell segmentation and recorded mean expression levels of C-terminally phosphorylated SMAD1/5 (pSMAD1/5) in every cell. Then, pSMAD1/5 signals were classified into "low" or "high", based on a visually defined threshold. Protein staining intensities quantified in every cell in three selected areas per sample, represented a total of 4 000 to 10 000 cells depending on the sample, and were plotted as percent of the total cell number. Orthotopic xenografts were stained using the diaminobenzidine substrate and Mayer's

hematoxylin for nuclear counterstain, imaged in Vectra-Polaris and analyzed by QuPath, https://qupath.github.io/ [6].

Immunoblotting

Total proteins were extracted in 20 mM Tris-HCl, pH 7.5, 1% Nonidet P-40, 150 mM NaCl, 10 mM EDTA, protease inhibitor Cømplete and PhosSTOP (Roche Diagnostics Scandinavia AB, Bromma, Sweden). Lysates were centrifuged at 12 000×*g* for 10 min at 4°C, and protein concentration in the cleared supernatants was measured (Bradford assay; Bio-Rad Laboratories Inc., Sundbyberg, Sweden). Equal amounts (20-40 µg) of denatured protein lysates were subjected to SDS-polyacrylamide gel electrophoresis, transferred to nitrocellulose in a wet unit (Bio-Rad Laboratories Inc., Sundbyberg, Sweden), blocked in 5% BSA or 5% skim milk, and filters were incubated with primary antibodies (Supplementary Table S1), horseradish peroxidase-conjugated secondary antibodies and developed using enhanced chemiluminescence assays (Merck/Millipore Stockholm, Sweden). Note that the antibody recognizing LHX2 is monoclonal and its epitope maps in the highly conserved middle exons, making it possible to recognize the two major isoforms of LHX2 derived by alternative promoter and alternative splicing (www.ensembl.org/Homo_sapiens/ENSG00000106689). All original immunoblots are presented as Supplementary files.

Spatial RNA sequencing

Two Group 4 PDXs (DMB006, RCMB029) were analyzed by NanoString GeoMx® Digital Spatial Profiler (DSP), according to the manufacturer's protocol (NanoString Technologies Inc., Seattle, WA, USA). Freshly cut FFPE sections were incubated with

RNA probes coupled to photocleavable oligonucleotide tags and pSMAD1/5 (BMP signaling), glial fibrillar acidic protein (GFAP, glial cell cytoskeleton) and SYTO13 (nucleic acid staining) serving as *in situ* markers. Eighteen regions of interest (ROIs) were selected based on pSMAD1/5 signal intensity quantification. Nine ROIs with pSMAD1/5 intensity higher than the median value of the 18 regions were called "pSMAD1/5-high" and nine with lower pSMAD1/5 intensity were called "pSMAD1/5-low". Photocleavable probes from individual ROIs were collected via UV exposure and subjected to library preparation (NanoString protocol, NanoString Technologies Inc., Seattle, WA, USA). High throughput sequencing was performed on the Illumina NovaSeq 6 000 platform (Illumina Inc., San Diego, CA, USA). Data processing in the DSP analysis server, including adaptor trimming, was followed by QC check, alignment and quantification. Probe QC was applied for targeted probes and negative controls. Low-count raw reads were excluded based on 1% filtering criteria, followed by count normalization using the third quartile (Q3) method (NanoString Technologies Inc., Seattle, WA, USA). A matrix of normalized counts for all 18 ROIs was generated (Supplementary Table S2), and differential expression analysis between pSMAD1/5-low and -high ROIs followed the limma package in R [7], and a robust fitting model with the false discovery rate (FDR) method to calculate the adjusted *p*-value. Supplementary Tables S2 and S3 list differentially expressed genes (FDR<0.05 and $\log_2$ fold-change ≥±1).

Cellular RNA sequencing

Total RNA from D283, MB002, D425, CHLA-01-MED and CHLA-01R-MED cell lines with or without BMP7 (100 ng/ml) treatment or from CHLA-01R-MED cells treated with DMSO (0.01% of total volume) or LDN193189 (0.2 μM) for 3 days, was isolated using

the PureLink RNA Mini kit (Thermo Fisher Scientific, Stockholm, Sweden, Cat#A21206). For library preparation, 500 ng of total RNA were extracted using ReliaPrep™ RNA Cell Miniprep System (Promega, Madison, WI, USA) and RNA integrity was assessed by Agilent-2100 Bioanalyzer (Agilent Technologies, Santa Clara, CA, USA). rRNA was depleted (RiboZero Gold treatment) and libraries were prepared with the TruSeq stranded total RNA Gold library kit of unique dual indexes (protocol#1000000040499, Illumina Inc. San Diego, CA, USA). The libraries were sequenced with 150 bp paired-ends in an S4 flow-cell with v1.5 sequencing chemistry running on a NovaSeq-6 000 platform (Illumina Inc., San Diego, CA, USA). Raw sequencing fastq files were trimmed, aligned against the human genome GRCh38, assessed for quality and quantified with the standardized RNA-seq pipeline of nf-core/rnaseq v1.4.2 [8]. Differential gene expression analysis was performed by DESeq2 Bioconductor package in R [9], with cut-off criteria of $\log_2$ fold-change ±2 and FDR<0.05. Data visualization was carried out in RStudio v1.4.1717 with R v4.0.5, and primary data are available at GEO (accession number GSE229150), with additional details provided in Supplementary Table S4.

Reverse transcription and real-time quantitative PCR (RT-qPCR)

Total RNA extracted from cells using TRIzol Reagent (Thermo Fisher Scientific, Stockholm, Sweden) according to the manufacturer, was quantified using NanoDrop 2 000 (Thermo Fisher Scientific, Stockholm, Sweden). Equal RNA amounts (0.5-1 μg) were reverse transcribed using the iScript cDNA synthesis kit (Bio-Rad Laboratories Inc., Sundbyberg, Sweden). RT-qPCR was performed on a Bio-Rad CFX96 cycler (Bio-Rad Laboratories Inc., Sundbyberg, Sweden) using the qPCRBIO SyGreen Master Mix (PCR Biosystems, London, UK) and primers as shown in Supplementary

Table S5. Target gene expression levels calculated by the standard curve method were normalized to the reference gene *GAPDH* levels.

Cleavage Under Targets & Release Using Nuclease (CUT&RUN) assays

CHLA-01R-MED and MB002 cells were cultured with 100 ng/ml BMP7 or vehicle for 3 days, and 2.5×10^5 cells/condition were dissociated into single-cell suspension through rigorous pipetting and treatment with trypsin and analyzed using the CUT&RUN Assay Kit (Cell Signaling Technology, #86652, Danvers, MA, USA), according to the manufacturer. Specific primers were designed surrounding the LHX2 consensus binding motif present within 1 kbp upstream from the transcription start sites (TSS) of the BMP receptor genes. Antibodies are listed in Supplementary Table S1, and primers for genomic DNA qPCR in Supplementary Table S5.

R2 data analysis

RNA expression profiles of normal cerebellum, MB and other (breast, cervix, colon, endometrium, kidney, lung, prostate, thyroid) patient samples were obtained and analyzed using R2: Genomics Analysis and Visualization Platform (http://r2.amc.nl). The MB data sets of Cavalli *et al.* (n=763) [10] or Pfister *et al*. (n=223) [11] were analyzed as indicated in the figures or figure legends. For other tumors, “XPO sampler” (Expression Project for Oncology) in R2 pre-set platform was used as a series of datasets. The dataset of Roth *et al.* (n=9) [12] was used to analyze normal cerebellum. Analysis of overall survival was performed using the Cavalli *et al.* patient data set [10] in R2 by Kaplan-Meier plots and log-rank tests.

Supplementary References

1. Ferrucci V, de Antonellis P, Pennino FP, Asadzadeh F, Virgilio A, Montanaro D, et al. Metastatic group 3 medulloblastoma is driven by PRUNE1 targeting NME1-TGF-β-OTX2-SNAIL via PTEN inhibition. Brain. 2018;141(5):1300-19.
2. Suryo Rahmanto A, Savov V, Brunner A, Bolin S, Weishaupt H, Malyukova A, et al. FBW7 suppression leads to SOX9 stabilization and increased malignancy in medulloblastoma. EMBO J. 2016;35(20):2192-212.
3. Valcourt U, Kowanetz M, Niimi H, Heldin C-H, Moustakas A. TGF-β and the Smad signaling pathway support transcriptomic reprogramming during epithelial-mesenchymal cell transition. Mol Biol Cell. 2005;16(4):1987-2002.
4. Korchynskyi O, ten Dijke P. Identification and functional characterization of distinct critically important bone morphogenetic protein-specific response elements in the Id1 promoter. J Biol Chem. 2002;277(7):4883-91.
5. Borgenvik A, Holmberg KO, Bolin S, Zhao M, Savov V, Rosen G, et al. Dormant SOX9-Positive Cells Facilitate MYC-Driven Recurrence of Medulloblastoma. Cancer Res. 2022;82(24):4586-603.
6. Bankhead P, Loughrey MB, Fernandez JA, Dombrowski Y, McArt DG, Dunne PD, et al. QuPath: Open source software for digital pathology image analysis. Sci Rep. 2017;7(1):16878.
7. Ritchie ME, Phipson B, Wu D, Hu Y, Law CW, Shi W, et al. limma powers differential expression analyses for RNA-sequencing and microarray studies. Nucleic Acids Res. 2015;43(7):e47.

8. Ewels PA, Peltzer A, Fillinger S, Patel H, Alneberg J, Wilm A, et al. The nf-core framework for community-curated bioinformatics pipelines. Nat Biotechnol. 2020;38(3):276-8.
9. Love MI, Huber W, Anders S. Moderated estimation of fold change and dispersion for RNA-seq data with DESeq2. Genome Biol. 2014;15(12):550.
10. Cavalli FMG, Remke M, Rampasek L, Peacock J, Shih DJH, Luu B, et al. Intertumoral Heterogeneity within Medulloblastoma Subgroups. Cancer Cell. 2017;31(6):737-54 e6.
11. Pfister S, Remke M, Benner A, Mendrzyk F, Toedt G, Felsberg J, et al. Outcome prediction in pediatric medulloblastoma based on DNA copy-number aberrations of chromosomes 6q and 17q and the MYC and MYCN loci. J Clin Oncol. 2009;27(10):1627-36.
12. Roth RB, Hevezi P, Lee J, Willhite D, Lechner SM, Foster AC, et al. Gene expression analyses reveal molecular relationships among 20 regions of the human CNS. Neurogenetics. 2006;7(2):67-80.

Supplementary Table S1: Primary Antibodies for immunoblot, CUT&RUN, multispectral immunofluorescence and immunohistochemistry.

Primary Antibodies for immunoblot and immunohistochemistry					
**Primary antibody for immunoblot**	**Manufacturer**	**Animal species**	**Dilution**		
β-actin	Santa Cruz Biotechnology, AC-15, sc-69879	Mouse, monoclonal	1:1,000		
SMAD1	Abcam, ab33902	Rabbit, monoclonal	1:1,000		
Phospho-SMAD1 (Ser463/465)/ Smad5 (Ser463/465)/ Smad9 (Ser465/467)	Cell Signaling Technology, D5B10	Rabbit, monoclonal	1:1,000		
ID1	Santa Cruz Biotechnology, Z-8, SC-427	Rabitt, polyclonal	1:1,000		
Flag M2	MERCK/Sigma-Aldrich, anti-Flag, M2, F1804	Mouse, monoclonal	1:1,000		
ACVR1/ALK2	LS Bio, (aa 80-129), LS-B6835	Rabitt, polyclonal	1:1000		
GFP	Santa Cruz Biotechnology, SC-99C	Mouse, monoclonal	1:1,000		
LHX2	Abcam, ab184337	Rabitt, monoclonal	1:1,000		
**Primary antibody for CUT&RUN**	**Manufacturer**	**Animal species**	**Dilution**		
LHX2	Abcam, ab184337	Rabitt, monoclonal	1:100		
Tri-Methyl-Histone H3 (Lys4) (C42D8)	Cell Signaling Technology, 9751	Rabbit monoclonal	1:100		
SMAD1	Abcam, ab33902	Rabbit, monoclonal	1:100		
**Primary antibody for multispectral immunofluorescence**	**Manufacturer**	**Animal species**	**Dilution**	**Antigen retrieval Buffer**	**Opal fluorophores**
Phospho-SMAD1/SMAD5 (Ser463, Ser465)	Thermo Fisher Scientific, 700047	Rabbit, monoclonal	1:100	pH6	570
**Primary antibody for immunohistochemistry**	**Manufacturer**	**Animal species**	**Dilution**	**Antigen retrieval Buffer**	
LHX2	Abcam, ab184337	Rabitt, monoclonal	1:100	pH9	
β-catenin	Becton Dickinson, BD610154	Mouse, monoclonal	1:100	pH6	
YAP1	Santa Cruz Biotechnology, SC-101199	Mouse, monoclonal	1:100	pH6	
SMAD1	Abcam, ab33902	Rabbit, monoclonal	1:100	pH6	
Phospho-SMAD1 (Ser463/465)/ Smad5 (Ser463/465)/ Smad9 (Ser465/467)	Cell Signaling Technology, D5B10	Rabbit, monoclonal	1:100	pH6	
ID3	Santa Cruz Biotechnology	Rabitt, polyclonal	1:100	pH9	

Supplementary Table S2: Spatial transcriptomics data table of QC reports and raw counts. Separate excel file.

Supplementary Table S3: Complete list of DEGs obtained from NanoString spatial transcriptomics data analysis. Separate excel file.

Supplementary Table S4: Complete list of all DEGs in cell lines stimulated with BMP7 or treated with LDN193189. Separate excel file.

Supplementary Table S5: Primer sequences (RT-qPCR, CUT&RUN).

Primer sequences used for RT-qPCR assays.

Gene Symbol	**Forward** (F) **and Reverse** (R) **Primers Sequences (5'- 3')**
hLHX2	F - GGGTCCTCCAGGTCTGGTT<br>R - GTTGGAGAGCTCCGAGGC
hACVR1	F - GTGAAGGTCTCTCCTGCGGTA<br>R - GCCATCGTTGATGCTCAGTGA
hSMAD1	F - GTCTGCATCAATCCCTACCAC<br>R - GCTCATTTTGTCCTAAGTTACGG
hSMAD4	F - CATCCTGGACATTACTGGCCA<br>R - CCTACCTGAACGTCCATTTCAA
hSMAD5	F - TTCCCTTATCTCCAAACAGCC<br>R - CATCAGGTGGCATATAGGCAG
hID1	F- GGACGAGCAGCAGGTAAACG<br>R- TGCTCACCTTGCGGTTCTG
hGAPDH	F - GGAGTCAACGGATTTGGTCGTA<br>R - GGCAACAATATCCACTTTACCA

Primer sequences used for CUT&RUN assays.

Gene Symbol	**Forward** (F) **and Reverse** (R) **Primers Sequences (5'- 3')**
hACVR1 primer 1 (intronic)	F - TGCTGGCTGGGATCACTTTT<br>R - TGCAGGTTTGCTTGAACACA
hACVR1 primer 2 (upstream)	F - GCCTCCTCTCTCCCCTCC<br>R - GCTGTGATTAACCAGGCAGC
hACVR2A-1	F - ATGCACAGATAAGACAAGAGTGG<br>R - ATGTGTAAGCCTTCCATTTTCTG
hACVR2A-2	F - GTATTTAGTGTGGGCTCCTCC<br>R - GGAGACCCAGATTAAATTGCTGA
hACVR2B	F - GGGAATCCAAGGCACAGAGG<br>R - TCCTGACCACTTTGCCCG
hBMPR1A-1	F - ATTCTAGTGGAGCAGAGGGG<br>R - CCAGGTAGGTCACAAGAGAGG
hBMPR1A-2	F - GTAACACATGGTTGTTAATTGC<br>R - CAAAGGCAGGAAAAGCAGACT
hBMPR1B-1	F - AAACTCACATTTCATTTTCGA<br>R - GGGCTTGAAGACACGGAAAT
hBMPR1B-2	F - TGCCAACGAATCTCTGTTTTCT<br>R - GACATTTCCCATAACTTGCCCA
hBMPR2-1	F - TAAAGCTAATGAATCAAAGCA<br>R - TTCCATAATTAGGGGTTGTAGCA
hBMPR2-2	F - CACTAGCAGCAACAACTTCTCA<br>R - GGCCAAGCAAAGTTTTACATGT
***LHX2*-region1_**	F- GGCTCCTCCTCCCACTCT<br>R - ACCGGGAGAATTGAAGAGGA
***LHX2*-region2**	F - CCTCTCCCCTCCCTCTTGG<br>R - CTTTTCTACAGCCGCGCC
***LHX2*-region3**	F - AAAACTCCAAACCCCGCG<br>R - GAGAGTCGAGATGGTGGGAG
***LHX2*-region4**	F - CCTACTCCAGTCGCCGAC<br>R - TCACCCTGAAGCCGCTAG

Supplementary figures

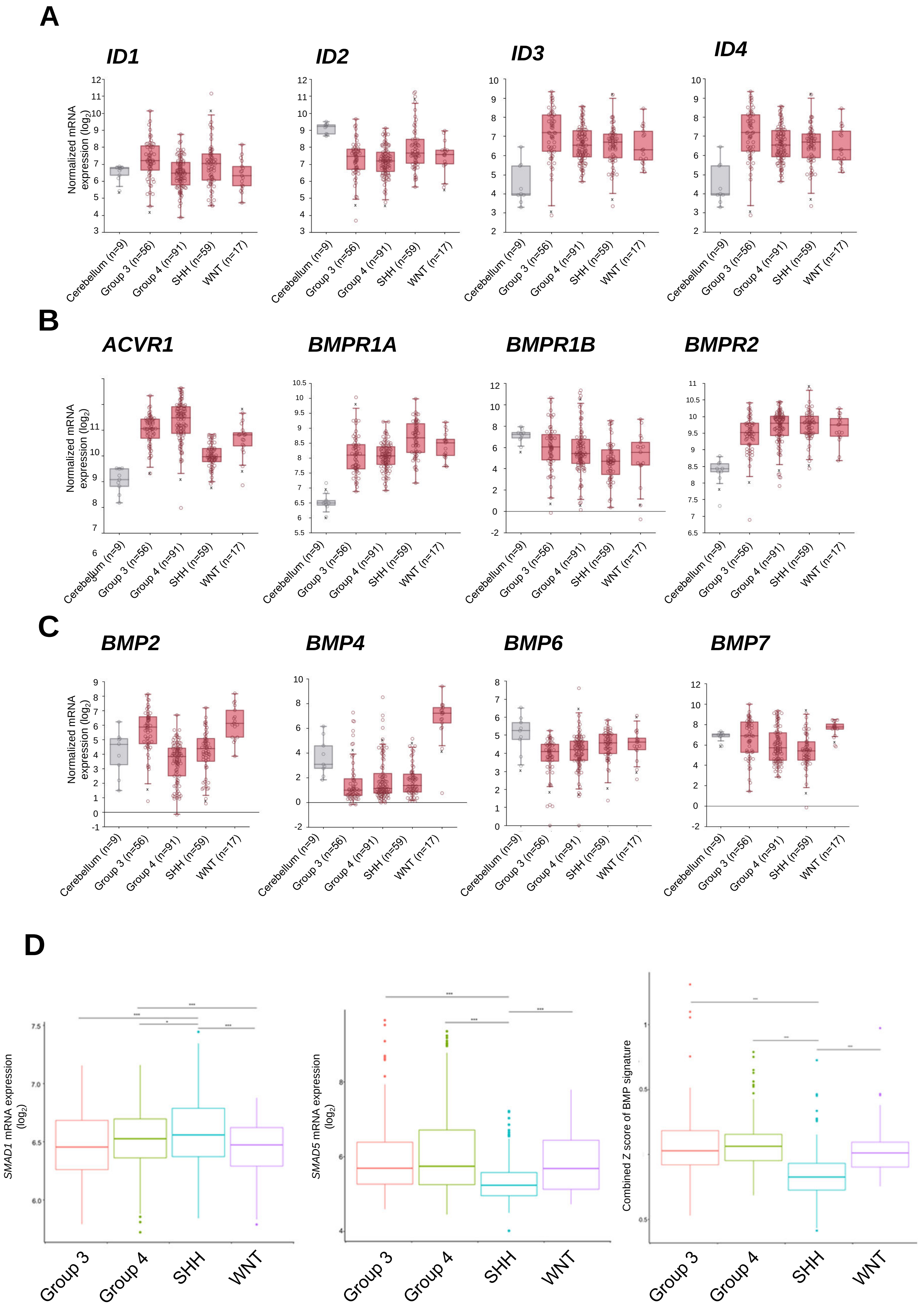

A
ID1
ID2
ID3
ID4
Normalized mRNA expression (log_2)
Cerebellum (n=9)
Group 3 (n=56)
Group 4 (n=91)
SHH (n=59)
WNT (n=17)
B
ACVR1
BMPR1A
BMPR1B
BMPR2
C
BMP2
BMP4
BMP6
BMP7
D
SMAD1 mRNA expression (log_2)
SMAD5 mRNA expression (log_2)
Combined Z score of BMP signature
Group 3
Group 4
SHH
WNT

Suppl Fig. S1

Supplementary Fig. S1. BMP signaling family gene expression: A-D mRNA expression in MB (Group 3, Group 4, SHH, WNT) and normal cerebellum were plotted from R2: Genomics Analysis and Visualization Platform (http://r2.amc.nl). **A** *ID1*, *ID2*, *ID3* and *ID4* mRNA expression. **B** mRNA expression of BMP signaling receptors. **C** mRNA expression of BMP family ligands. **D** *SMAD1*, *SMAD5* and combined Z-scores of core BMP signaling genes assessed in MB tumor samples. The data set of Pfister *et al*. [11] and Roth *et al.* [12] were used for the analysis of MB and normal cerebellum, respectively (A-C). The data set of Cavalli *et al*. [10] was used in panel (D). The numbers (n) of samples per group are indicated in each graph. Horizontal lines indicate the median, whiskers indicate the SEM and individual data points are shown. In panel (D), statistical significance is indicated (*p<0.05, **p<0.01, ***p<0.001) based on Wilcoxon rank sum test.

A

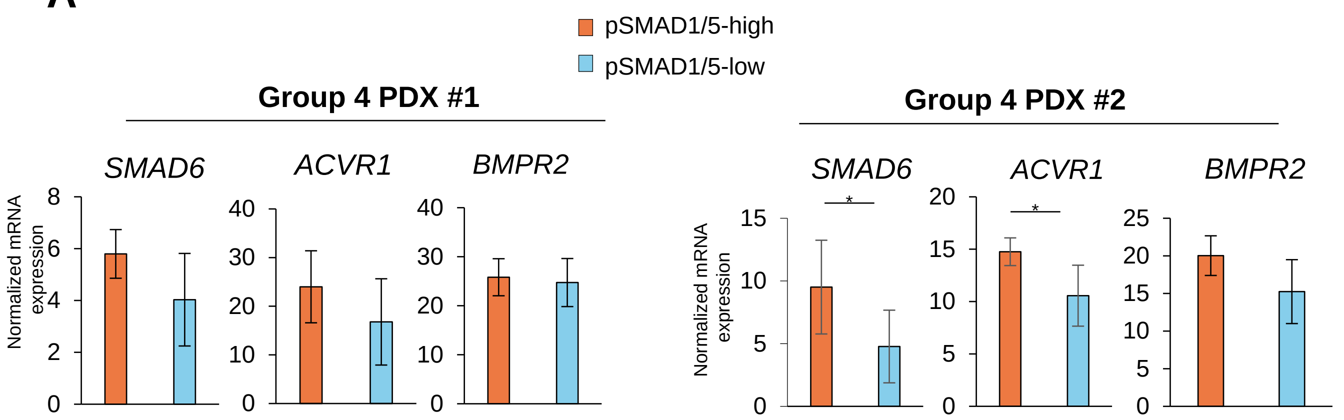

B

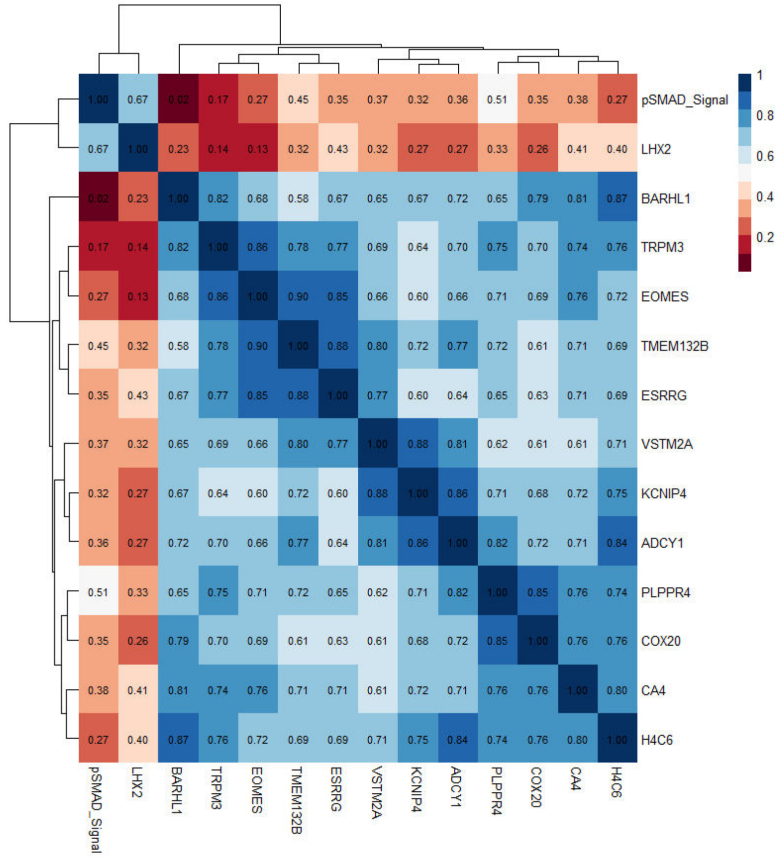

C

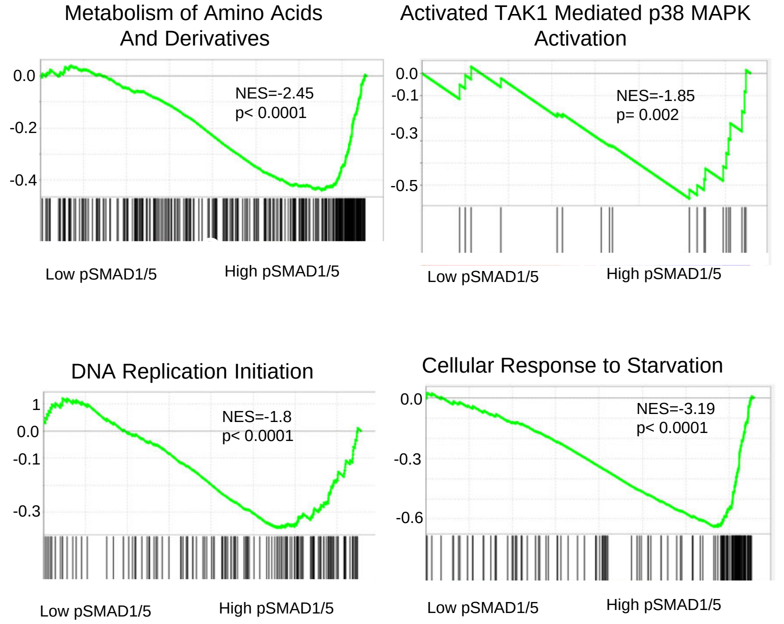

Suppl Fig. S2

Supplementary Fig. S2. Spatial transcriptomic analysis of Group 4 MB PDXs: A Normalized mRNA expression of *SMAD6*, *ACVR1* and *BMPR2* in pSMAD1/5-low and -high tumor cells from the two group 4 PDXs. Mean values with SEM derived from the 9 ROIs in each group are graphed. *p<0.05, comparing the two conditions (derived using the Wilcoxon rank sum test). **B** Correlation heatmap indicates the correlation (r values between 0 and 1 in the colored scale) between pSMAD1/5 intensity and the top 13 DEGs from the spatial transcriptomic analysis. Note the prominent correlation of *LHX2*. **C** Enrichment plots showing significantly enriched gene sets in pSMAD1/5-low tumor cells. Gene set enrichment analysis was performed using the Reactome database.

A

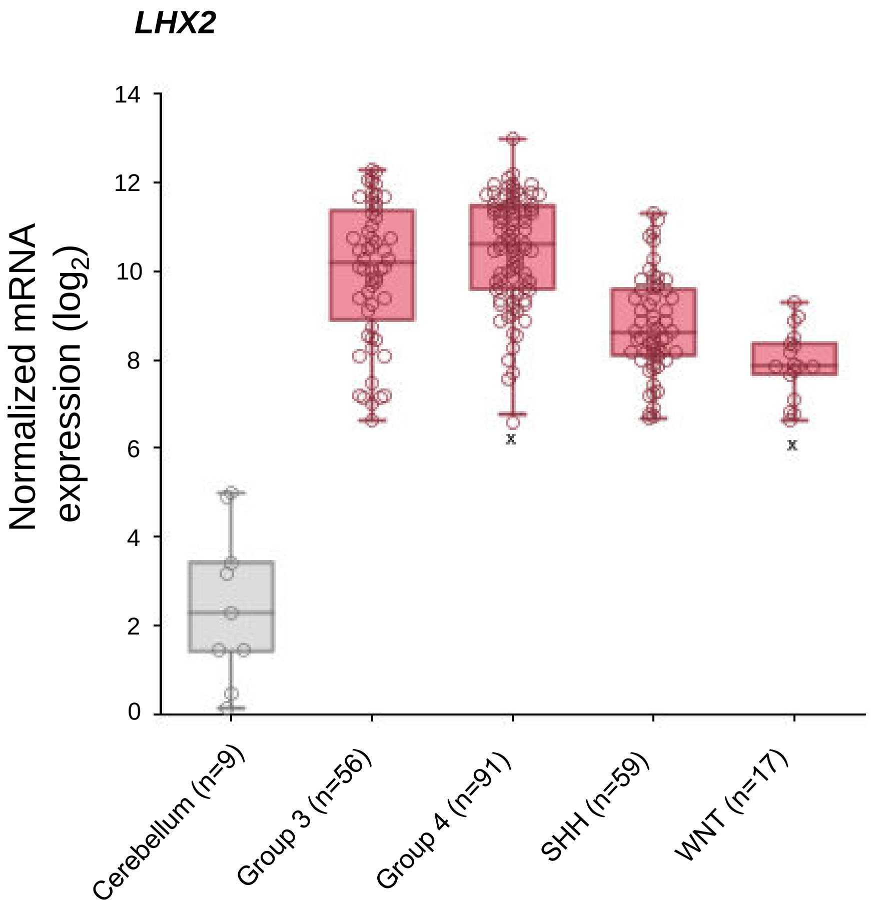

B

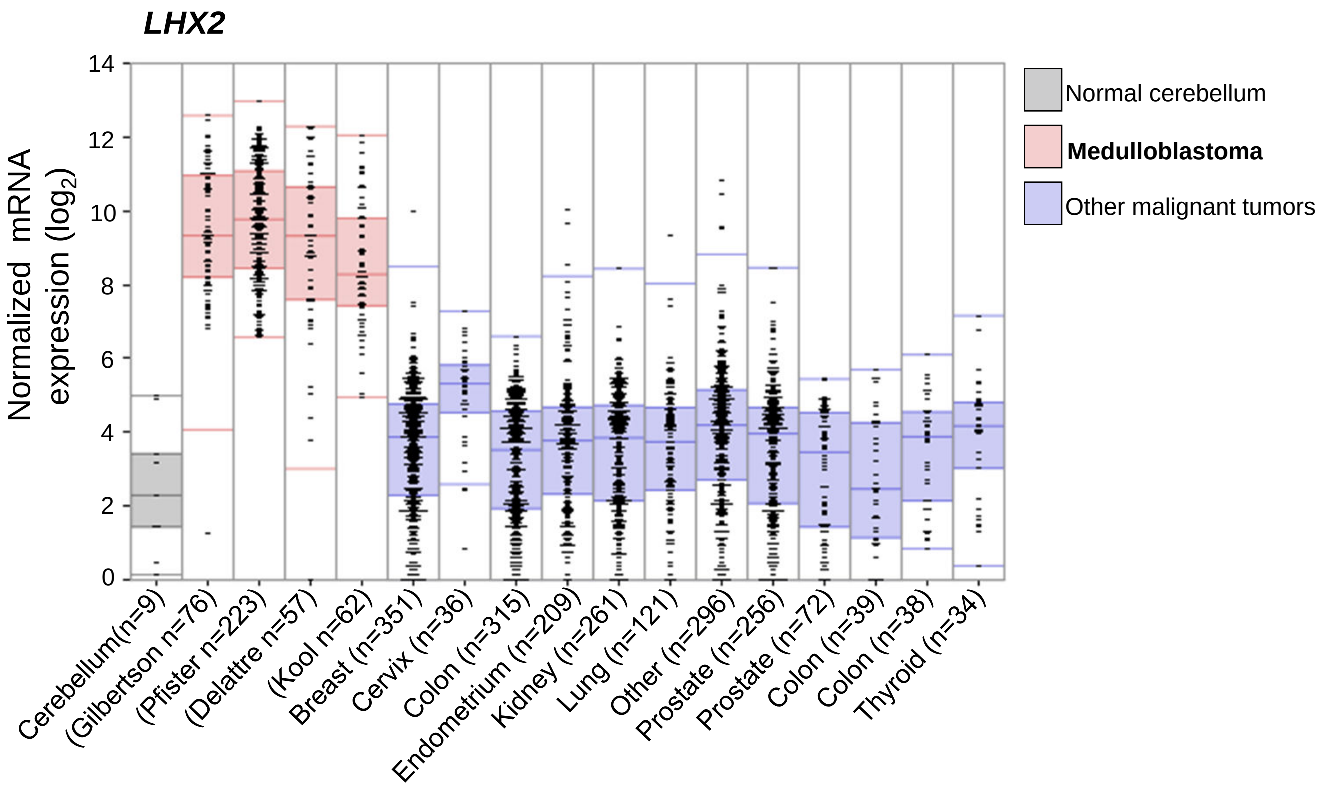

Suppl Fig. S3

Supplementary Fig. S3. *LHX2* is highly expressed in medulloblastoma: A, **B** *LHX2* mRNA expression in MB and normal cerebellum were plotted from R2: Genomics Analysis and Visualization Platform (http://r2.amc.nl). The data set of Roth *et al*. [12] was used for the analysis of normal cerebellum. The data set of Pfister *et al*. [11] was used for the analysis of MB and the data sets represented in the R2 platform were used for all other tumors (A). Four MB datasets were used as indicated in the graph (B). Numbers (n) of samples are indicated in the graphs. Horizontal lines indicate the median, whiskers indicate the SEM and individual data points are shown.

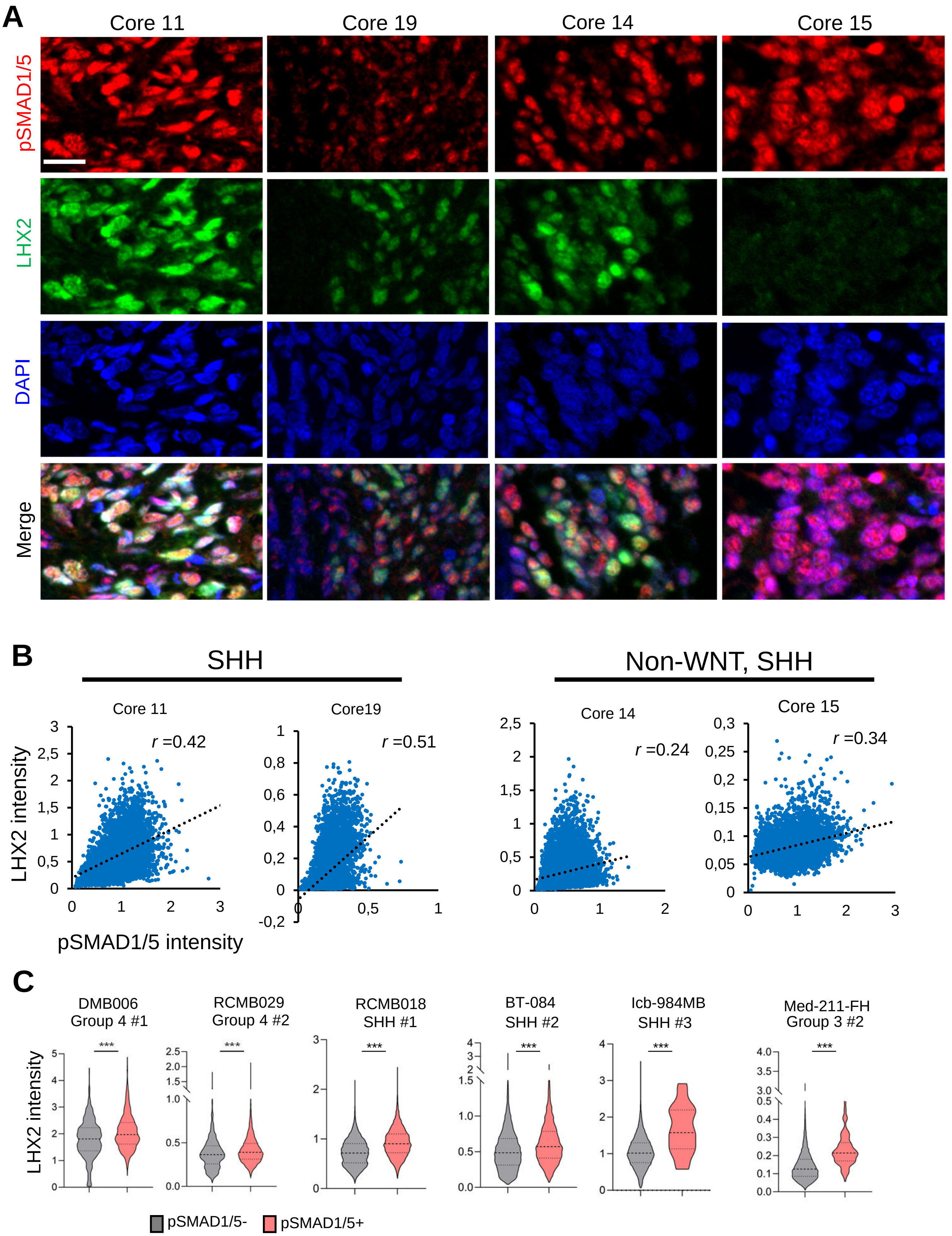
A
Core 11
Core 19
Core 14
Core 15
pSMAD1/5
LHX2
DAPI
Merge
B
SHH
Non-WNT, SHH
Core 11
Core19
Core 14
Core 15
r =0.42
r =0.51
r =0.24
r =0.34
LHX2 intensity
pSMAD1/5 intensity
C
DMB006
Group 4 #1
RCMB029
Group 4 #2
RCMB018
SHH #1
BT-084
SHH #2
Icb-984MB
SHH #3
Med-211-FH
Group 3 #2
***
LHX2 intensity
pSMAD1/5-
pSMAD1/5+

Suppl Fig. S4

Supplementary Fig. S4. LHX2 protein co-expression in pSMAD1/5-positive MB cells: A Representative images of 4 cores from the human MB TMA immunostained for pSMAD1/5 and LHX2. Scale bar: 10 µm. **B** Correlation analysis of pSMAD1/5 and LHX2 immunostaining signals from the 4 selected cores shown in panel (A), with corresponding r values. Each dot represents one cell in the tumor core section (core 11, n=5 170; core19, n=9 004; core 14, n=9 096; core 15, n=5 827). **C** Violin plots of LHX2 staining signals in pSMAD1/5 (positive or negative) cells in the PDX tumor samples of Fig. 1. Horizontal dotted lines indicate the median and corresponding upper and lower quartiles (DMB006, n=748 and 2 098; RCBM029, n=1 016 and 10 785; RCMB018, n=9 466 and 4 830; BT-084, n=5 685 and 3 082; Icb-984MB, n=8 790 and 180; Med-211-FH, n=9 736 and n=181, for pSMAD1/5-negative and -positive cells, respectively). ****p*<0.001 compared with control (two-tailed paired Student's t-test).

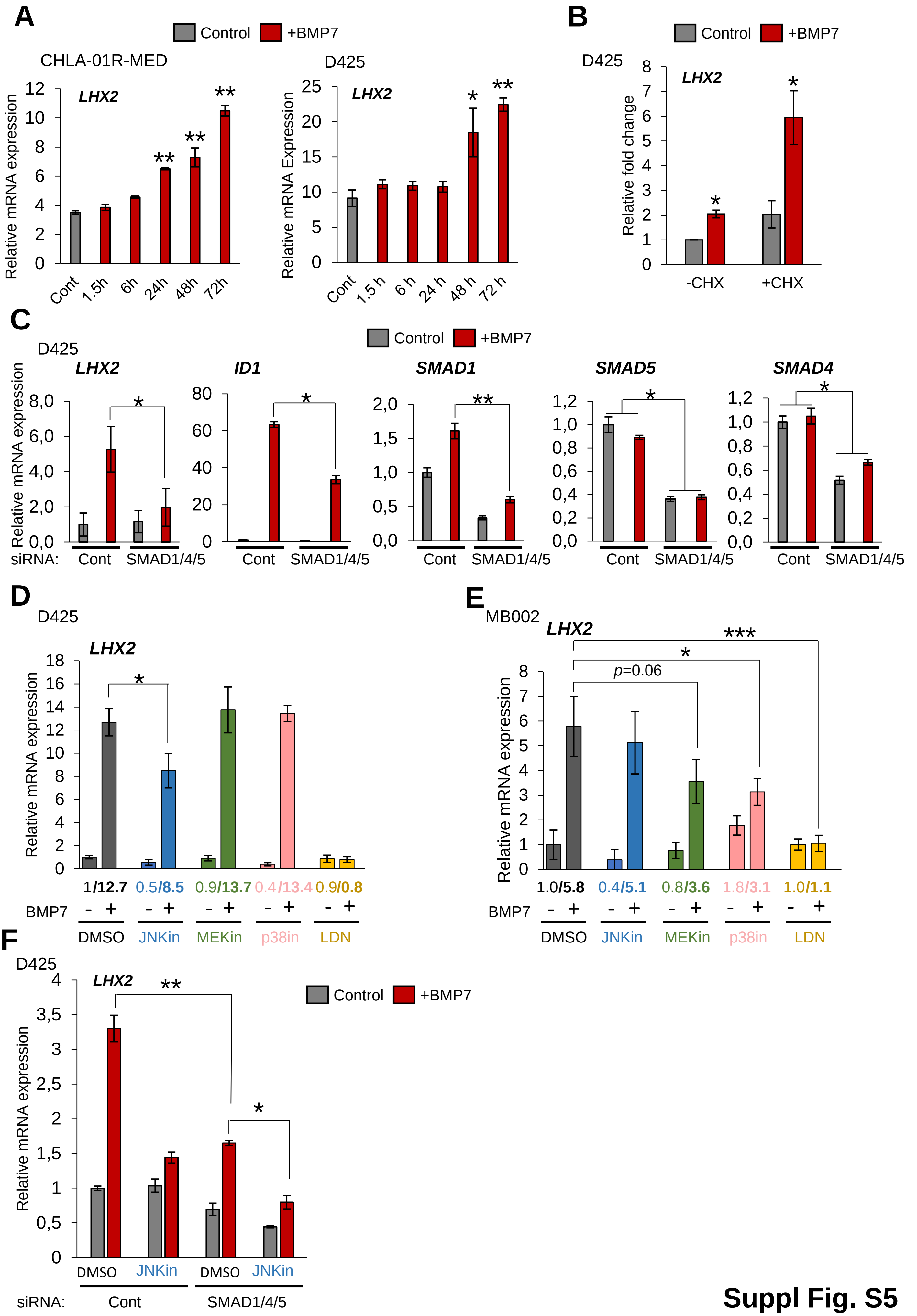
A
Control
+BMP7
CHLA-01R-MED
LHX2
Relative mRNA expression
Cont
1.5h
6h
24h
48h
72h
D425
LHX2
Relative mRNA Expression
Cont
1.5 h
6 h
24 h
48 h
72 h
B
D425
LHX2
Relative fold change
-CHX
+CHX
C
D425
LHX2
ID1
SMAD1
SMAD5
SMAD4
Relative mRNA expression
siRNA:
Cont
SMAD1/4/5
D
D425
LHX2
Relative mRNA expression
1/12.7
0.5/8.5
0.9/13.7
0.4/13.4
0.9/0.8
BMP7
DMSO
JNKin
MEKin
p38in
LDN
E
MB002
LHX2
p=0.06
Relative mRNA expression
1.0/5.8
0.4/5.1
0.8/3.6
1.8/3.1
1.0/1.1
BMP7
DMSO
JNKin
MEKin
p38in
LDN
F
D425
LHX2
Relative mRNA expression
DMSO
JNKin
DMSO
JNKin
siRNA:
Cont
SMAD1/4/5

Suppl Fig. S5

Supplementary Fig. S5. LHX2 gene regulation via SMAD and MAP-kinase signaling: A *LHX2* mRNA expression in CHLA-01-MED and D425 MB cells after treatment with BMP7 (100 ng/ml) over a 3-day period. **B** *LHX2* mRNA expression in D425 MB cells after treatment with BMP7 (100 ng/ml) for 72 h in the absence or presence of 40 µg/ml cycloheximide (CHX). **C-E** mRNA expression of the indicated genes in MB cell lines D425 (C, D) and MB002 (E) was measured by RT-qPCR 3 days after treatment with BMP7 (100 ng/ml). The results were normalized to *GAPDH* levels. The cells were transiently transfected with control (Cont) or specific siRNAs targeting *SMAD1*, *SMAD4* and *SMAD5* (C). Alternatively (D, E), cells were incubated with DMSO or 10 µM of each indicated inhibitor (in), whose specifications are given in the method. In panels D and E, the fold-expression level relative to the DMSO control without BMP7 is also reported numerically. **F** *LHX2* mRNA expression in MB D425 cells was measured by RT-qPCR 3 days after treatment with BMP7 (100 ng/ml) in the absence or presence of the same JNKin in cells transfected with control (Cont) or SMAD-specific triple siRNA. Representative data are presented as mean values ± SD of three biological replicates. *$p<0.05$, **$p<0.01$, ***$p<0.001$ compared with control (two-tailed paired Student's t-test).

A

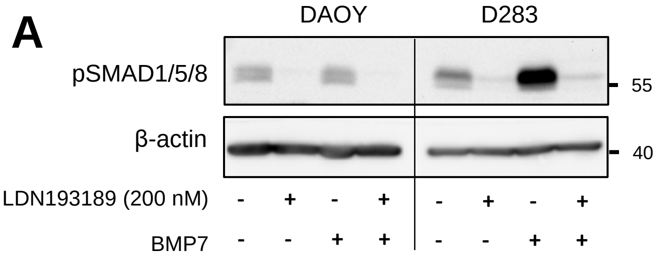

B

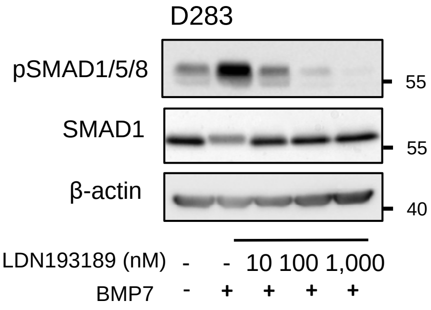

C

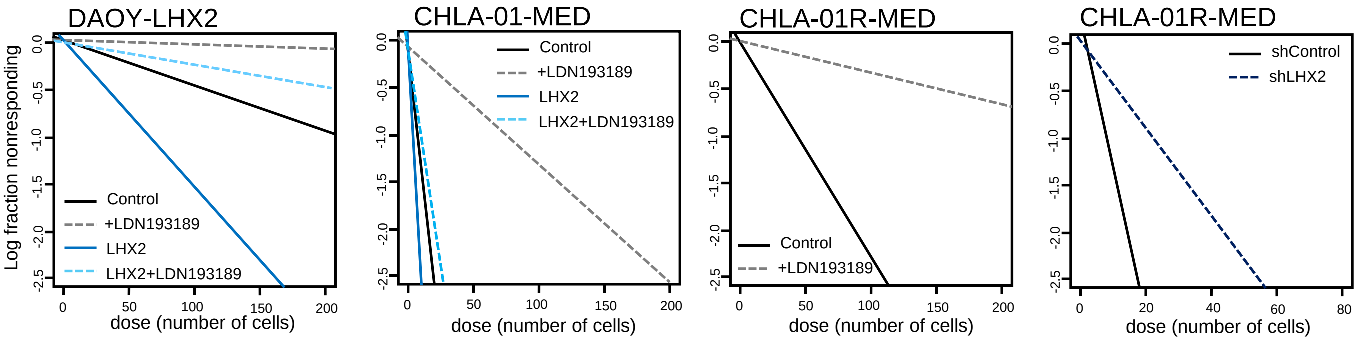

Condition	1/(Stem Cell Frequency)
Control	209
+LDN193189	2,281
3×Flag-LHX2	65
3×Flag-LHX2 +LDN193189	402

Condition	1/(Stem Cell Frequency)
Control	25
+LDN193189	233
3×Flag-LHX2	12
3×Flag-LHX2 +LDN193189	30

Condition	1/(Stem Cell Frequency)
Control	49
+LDN193189	303

Condition	1/(Stem Cell Frequency)
shControl	6.6
shLHX2#1	22

D

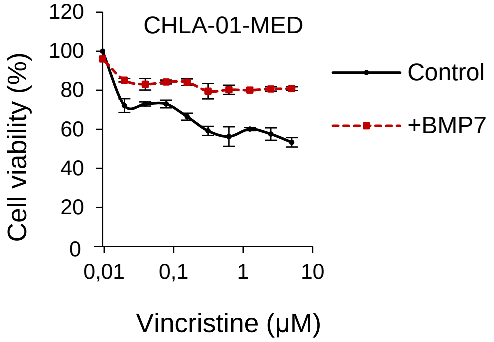

Suppl Fig. S6

Supplementary Fig. S6. BMP signaling inhibition and LHX2 impact on MB tumor-sphere formation: A DAOY and D283 cells were pre-treated with 0.2 µM LDN193189 or DMSO (as a control for each panel) for 30 min. Then cells were stimulated with 100 ng/ml BMP7 or vehicle for 2 h, and phosphorylated SMAD1/5/8 (pSMAD1/5/8) protein and β-actin (loading control) were analyzed by immunoblotting. **B** D283 cells were pre-treated with LDN193189 in a serial dilution (10, 100, 1 000 nM). Then the cells were stimulated with 100 ng/ml BMP7 for 2 h, and pSMAD1/5/8, SMAD1 and β-actin (loading control) were analyzed by immunoblotting. Molecular mass markers (kDa) are shown and original images are in Supplementary Fig. S12. **C** ELDA of the effects of LHX2 expression and LDN193189 treatment on tumor-sphere-forming frequency. The number of wells devoid of tumor-spheres (fraction nonresponding) was plotted against the number of plated cells per well. Steeper slopes indicate higher tumor-sphere-forming cell (stem cell) frequencies. The table indicates the average stem cell frequency per condition, derived from 2 biological replicates, each with 8 technical replicates, which are visualized as bar graphs in Fig. 4J, K. **D** CHLA-01-MED cells pre-treated with vehicle (Control) or 100 ng/ml BMP7 for 5 days were treated with vincristine in a serial dilution for 24 h. Cell viability was analyzed by PrestoBlue and mean values from 3 biological replicates, each with technical triplicates and corresponding SD are plotted as percent viability relative to the control (100%).

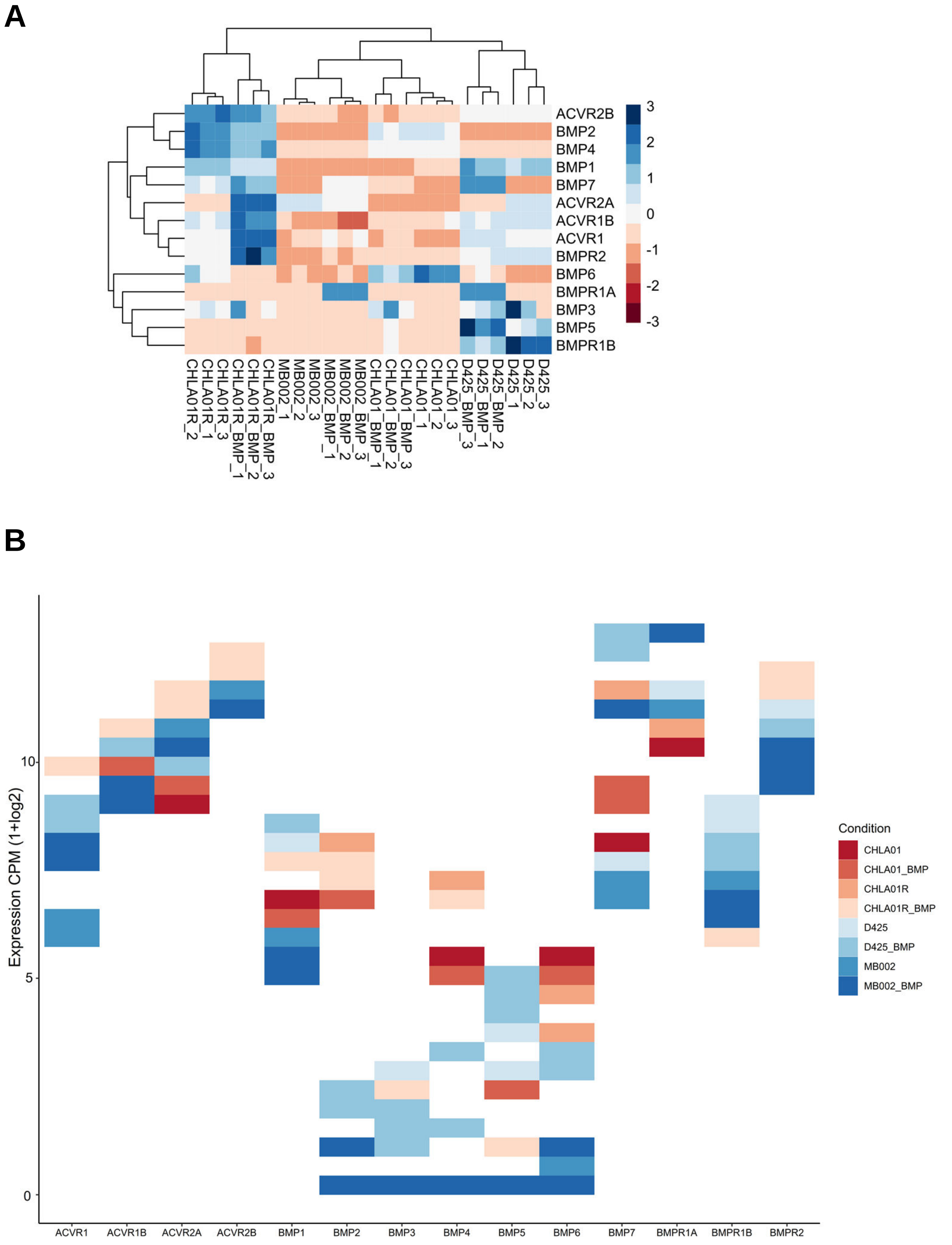
A
ACVR2B
BMP2
BMP4
BMP1
BMP7
ACVR2A
ACVR1B
ACVR1
BMPR2
BMP6
BMPR1A
BMP3
BMP5
BMPR1B
3
2
1
0
-1
-2
-3
CHLA01R_2
CHLA01R_1
CHLA01R_3
CHLA01R_BMP_1
CHLA01R_BMP_2
CHLA01R_BMP_3
MB002_1
MB002_2
MB002_3
MB002_BMP_1
MB002_BMP_2
MB002_BMP_3
CHLA01_BMP_1
CHLA01_BMP_2
CHLA01_BMP_3
CHLA01_1
CHLA01_2
CHLA01_3
D425_BMP_3
D425_BMP_1
D425_BMP_2
D425_1
D425_2
D425_3
B
Expression CPM (1+log2)
0
5
10
ACVR1
ACVR1B
ACVR2A
ACVR2B
BMP1
BMP2
BMP3
BMP4
BMP5
BMP6
BMP7
BMPR1A
BMPR1B
BMPR2
Condition
CHLA01
CHLA01_BMP
CHLA01R
CHLA01R_BMP
D425
D425_BMP
MB002
MB002_BMP

Suppl Fig. S7

Supplementary Fig. S7. BMP signaling pathway gene expression upon BMP7 stimulation in MB cell lines: A, B The indicated 14 BMP signaling genes were selected from the RNA-seq data from the 4 MB cell lines stimulated with BMP7 (Fig. 3). Their expression values relative to the cell line condition are shown in a heatmap (A) or a bar graph (B). Note that for reasons of inclusion during bioinformatic analysis, the BMP1 (tolloid-like and pro-collagen protease) is also included.

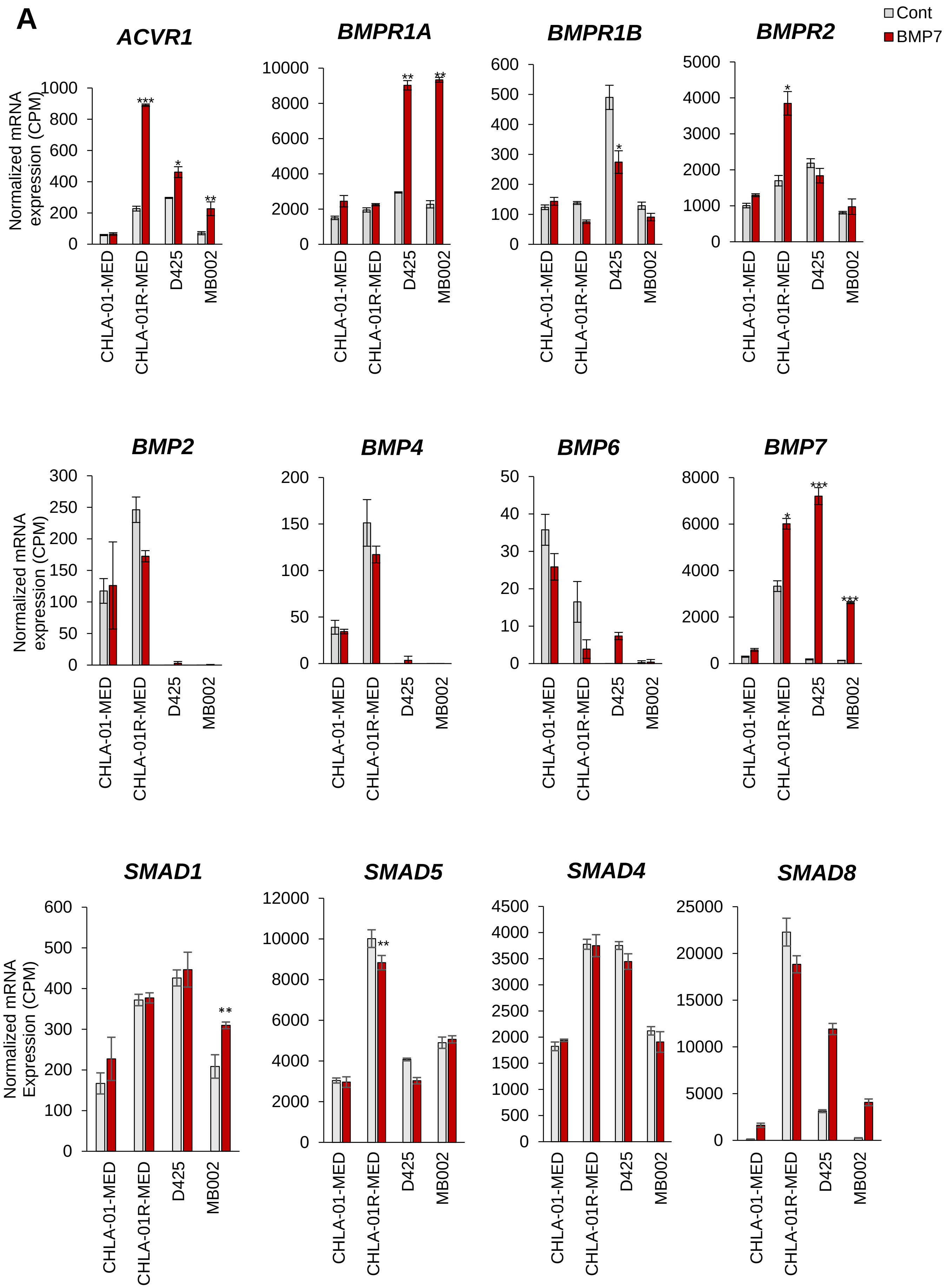
A
Cont
BMP7
ACVR1
BMPR1A
BMPR1B
BMPR2
BMP2
BMP4
BMP6
BMP7
SMAD1
SMAD5
SMAD4
SMAD8
Normalized mRNA expression (CPM)
Normalized mRNA Expression (CPM)
CHLA-01-MED
CHLA-01R-MED
D425
MB002

Suppl Fig. S8

B

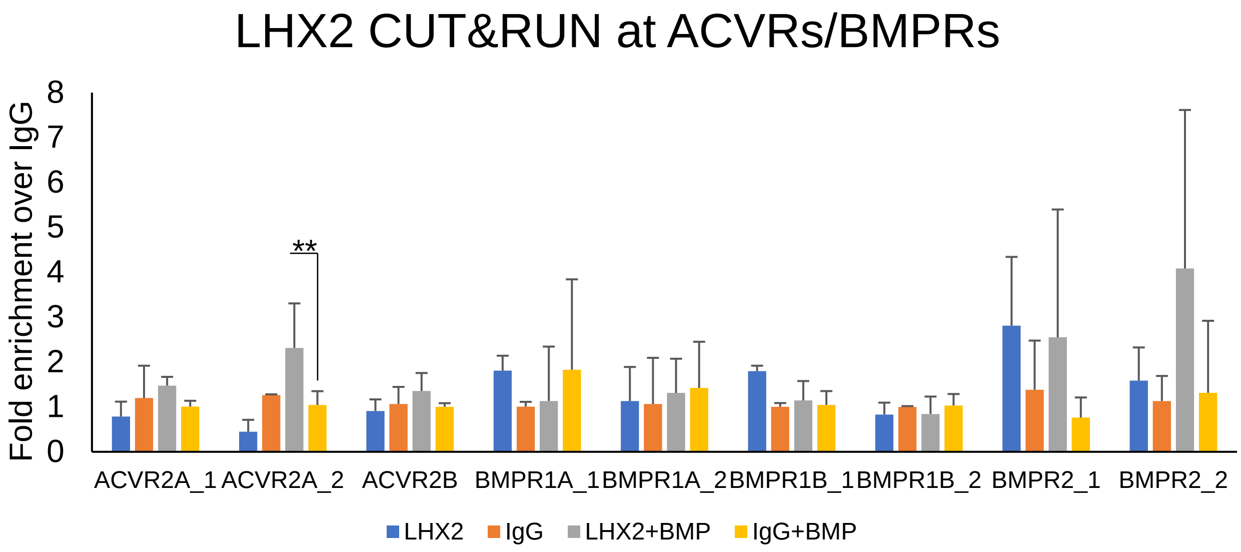

Suppl Fig. S8

Supplementary Fig. S8. Expression of BMP signaling genes and association of LHX2 on BMP receptor genes: A Expression of BMP receptor, ligand and SMAD mRNAs upon BMP7 stimulation in the indicated MB cell lines after stimulation for 3 days with 100 ng/ml BMP7 or vehicle (Cont) and expression analyzed by RNA-seq presented in Fig. 3 and plotted in bar-graphs with mean values from biological triplicates with SD and significance based on false discovery rate (FDR) (*$p<0.05$, **$p<0.01$, ***$p<0.001$) assessed by two-tailed paired Student's t-test. **B** CUT&RUN assay in CHLA-01R-MED cells, treated with 100 ng/ml BMP7 (BMP) or vehicle (-) for 3 days. Antibody against LHX2 was used. qPCR data for the 5 BMP receptor genes (with two independent gene locations amplified for the 4 out of 5 receptor genes; the second ACVR2B gene primer set never gave reliable qPCR reads) were normalized to the total amount of input chromatin and shown as fold-enrichment relative to IgG (negative control) for each condition. Data shown as the mean ± SD are representative, each with three technical replicates and 2 biological replicates. No significant differences were measured between the conditions, except for ACVR2A_2 (**$p<0.01$) assessed by one-way ANOVA with Tukey test. Primer sequences are shown in Supplementary Table S5.

A

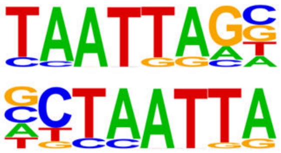

B

Annotation	Number of sites
3' UTR	13,056
Promoter-TSS	10,369
TTS	12,651
Intron	492,632
exon	6,153
intergenic	504,096
non-coding	5,280

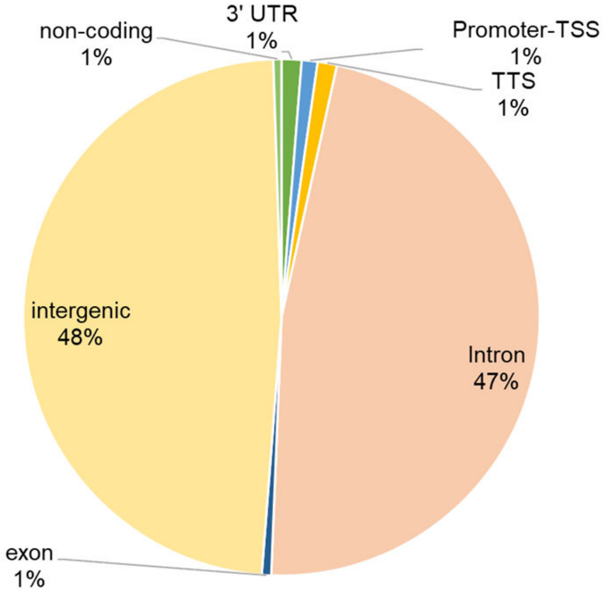

C

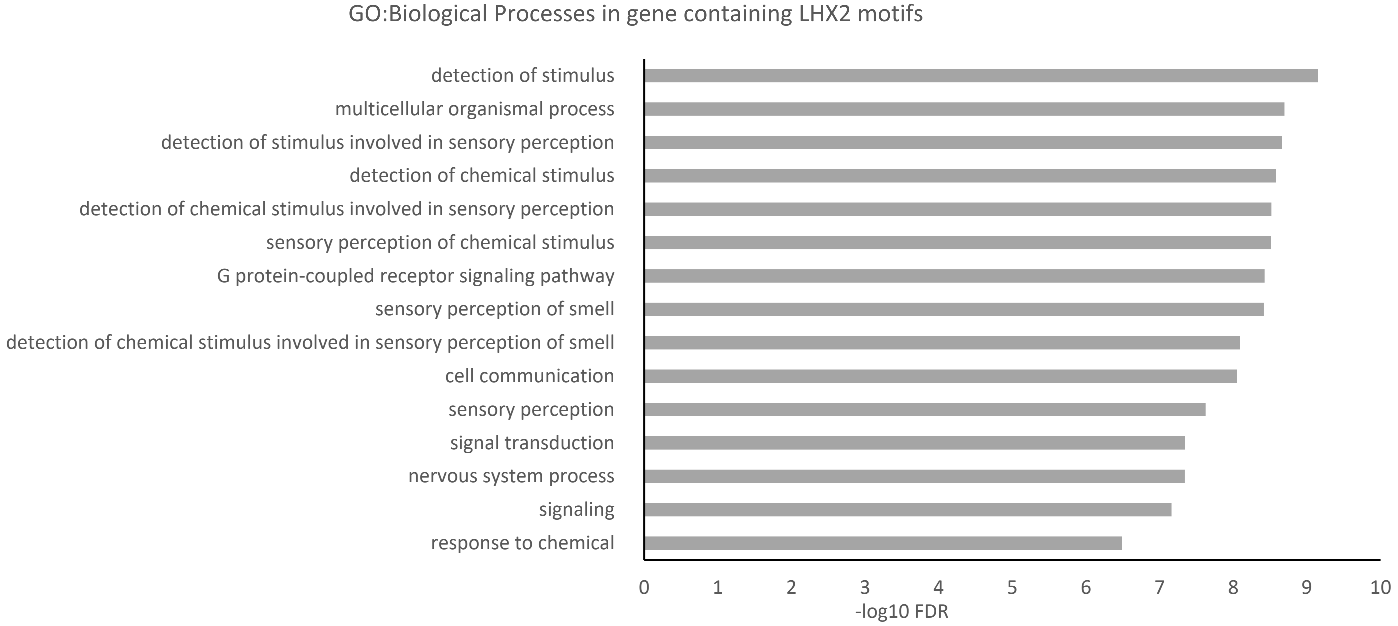

D

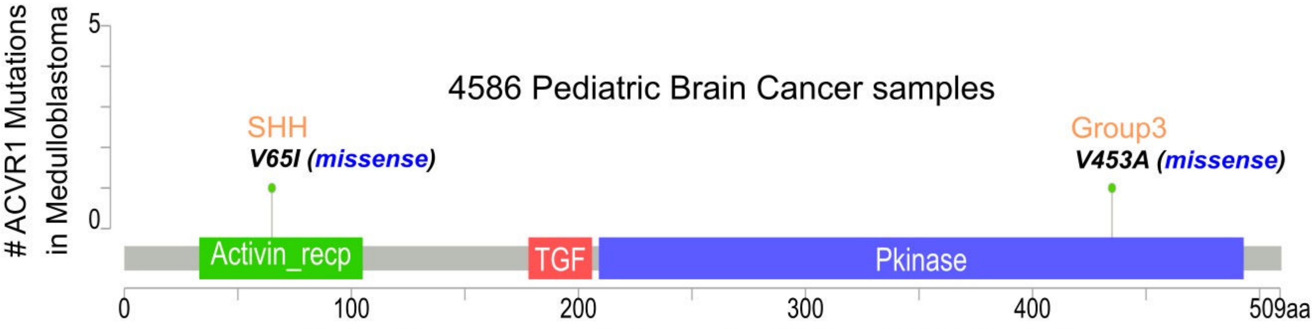

Suppl Fig. S9

Supplementary Fig. S9. LHX2 motif localization across the human genome and *ACVR1* genetics: A The consensus LHX2 binding motif retrieved from all available genomic and public databases, represented as a DNA sequence logo. **B** Table and corresponding pie-chart listing all consensus LHX2 binding motifs across the human genome, represented based on functionally annotated genomic regions (TSS, transcription start site; UTR, untranslated region). **C** Gene ontology biological processes corresponding to the unique genes harboring consensus LHX2 motifs within 1 kbp from their TSS. **D** Diagrammatic representation of the ACVR1 protein with its characteristic domains (extracellular activin receptor homology domain (Activin_recp), TGFβ family intracellular juxtamembrane phospho-acceptor peptide (TGF), intracellular protein kinase (Pkinase) domain, and its amino acid numbers plotted against the number of published mutations in MB patients. Two reported missense mutations and their location are shown. The data are derived from a provisional dataset that includes 4 586 samples of pediatric brain tumors. The two indicated mutations lack functional annotation.

A

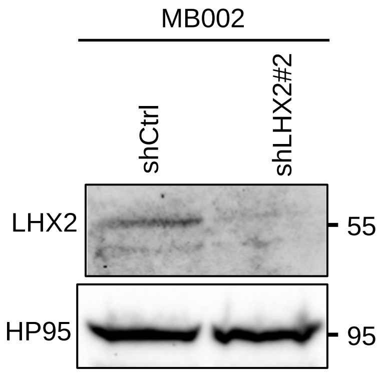

B

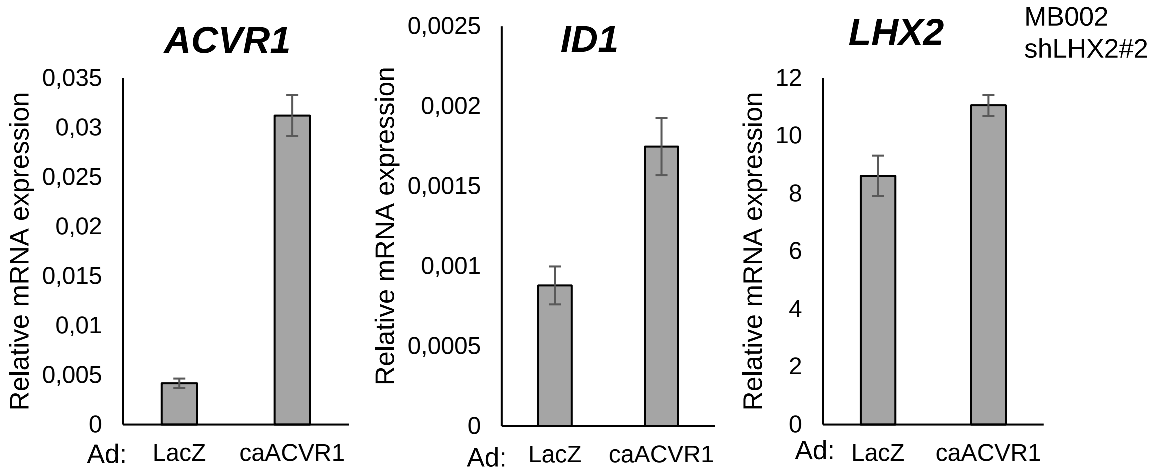

C

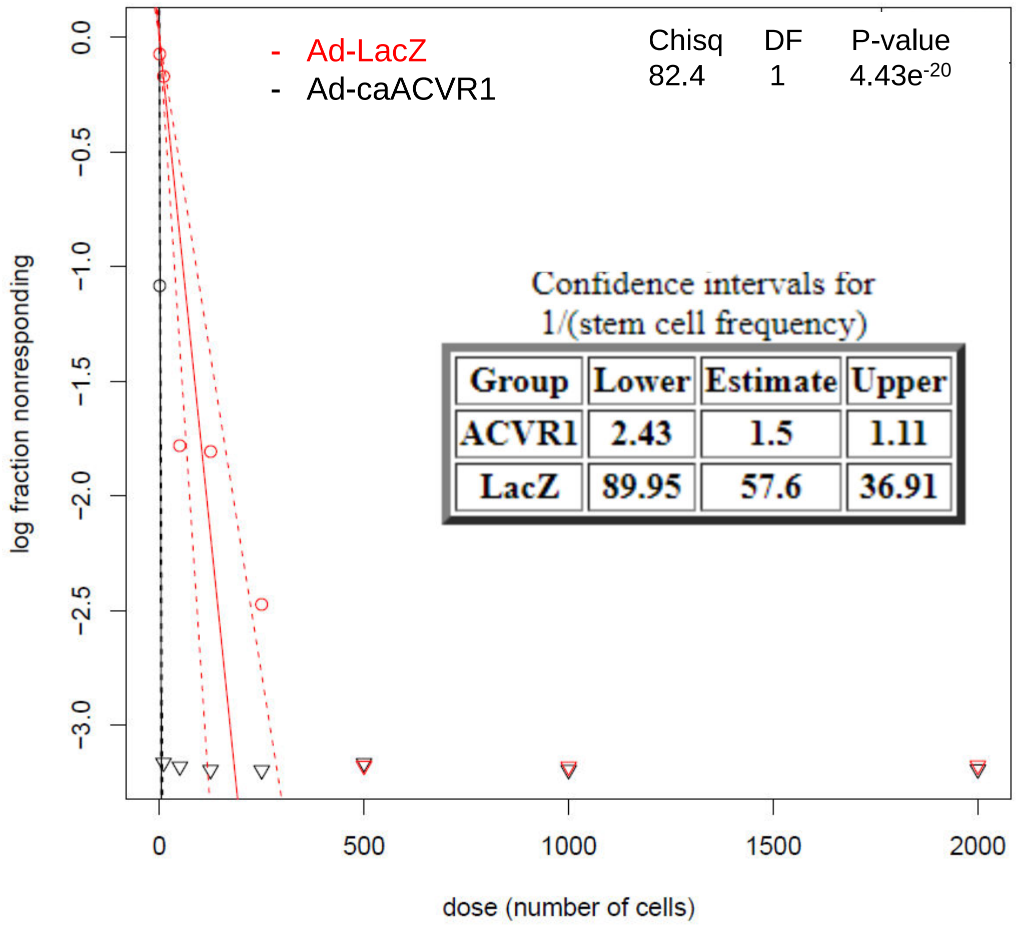

Group	Lower	Estimate	Upper
ACVR1	2.43	1.5	1.11
LacZ	89.95	57.6	36.91

Suppl Fig. S10

Supplementary Fig. S10. Rescue of ACVR1 expression in MB002 cells carrying stable LHX2 silencing: A Representative immunoblot of LHX2 and HP95 (loading control) in MB002 cell pools stably expressing control (shCtrl) or specific (shLHX2#2) shRNAs. Molecular mass markers (kDa) are shown. For original images, see Supplementary Fig. S12. **B** *ACVR1*, *ID1* and *LHX2* mRNA expression analyzed by RT-qPCR in MB002 cell pools stably expressing the shLHX2#2 vector, as indicated in panel (A). Cells were transiently infected with adenoviral vectors (Ad) expressing control LacZ or constitutively active (ca) ACVR1 (moi 5) and RNAs were isolated 3 days post-infection for expression analysis. Data shown are representative, each with three technical replicates from 4 biological replicates. **C** ELDA of the effects of caACVR1 expression on tumor-sphere-forming frequency. The number of wells devoid of tumor-spheres (fraction nonresponding) was plotted against the number of plated cells per well. Steeper slopes indicate higher tumor-sphere-forming cell (stem cell) frequencies. The table indicates the average stem cell frequency per condition, derived from 2 biological replicates, each with 8 technical replicates, and the result of a chi-square assay with 1 degree of freedom (DF) and associated *p*-value are reported. Corresponding SD are not shown as the ELDA software generates only upper and lower limits of the measurements.

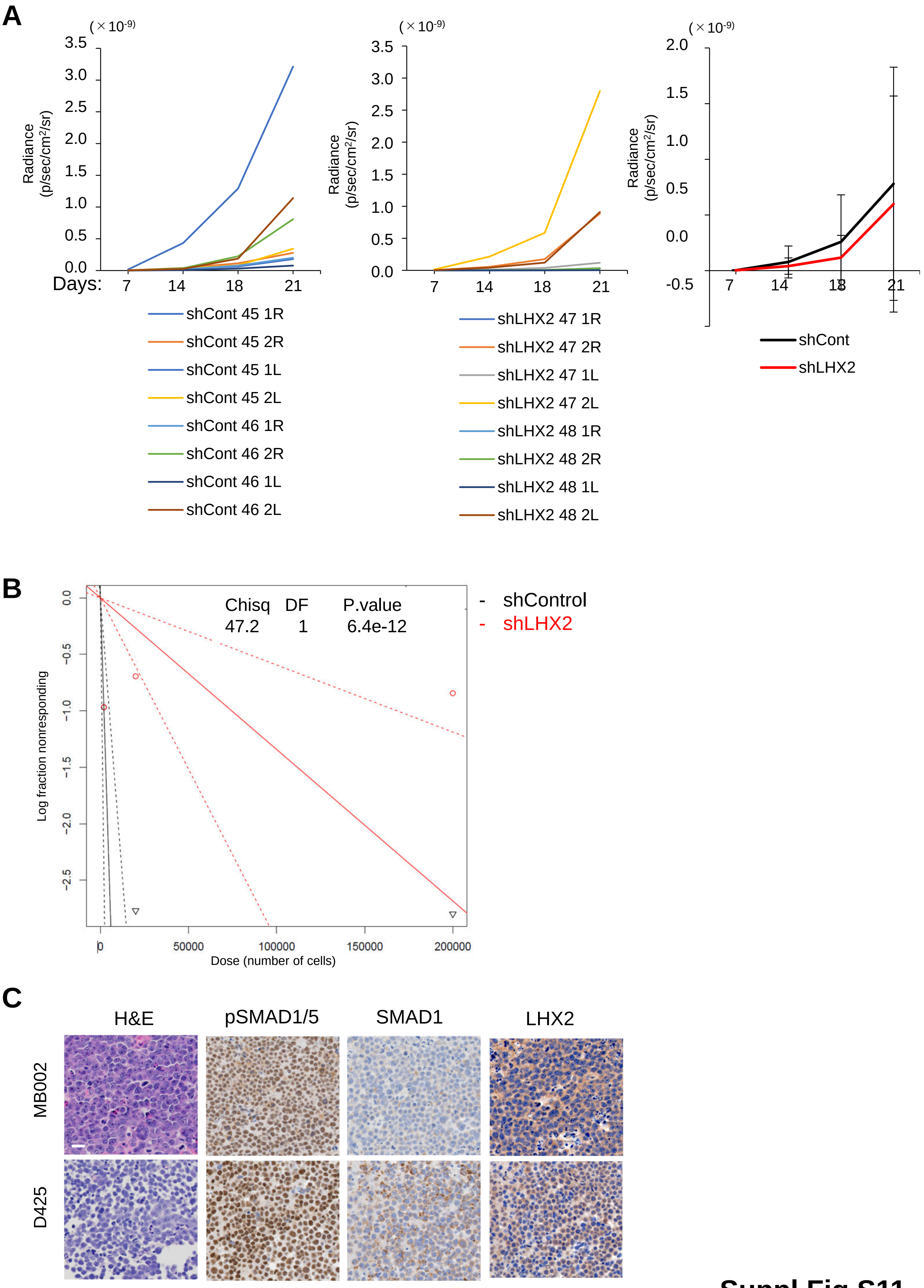

Suppl Fig.S11

Supplementary Fig. S11. LHX2 positively contributes to MB tumor propagation: **A** Growth curves of the primary orthotopic tumors. *In vivo* bioluminescence imaging of orthotopic xenografts (in 8 mice in total) generated by 2×10^4 CHLA-01R-MED cells carrying control (shCont) or LHX2-specific (shLHX2) shRNAs, and imaged every 4 days post-transplantation. The luminescence scale is shown in photons (p) per sec per cm^2 per steradian (sr). The diagram to the right shows average growth curves calculated from the data points of the 8 mice per biological condition (shCont, shLLHX2) with associated SD. **B** ELDA of the effect of LHX2 silencing on tumor-initiating frequency in mice. The number of animals devoid of tumors (fraction nonresponding) was plotted against the number of transplanted cells per mouse. Steeper slopes indicate higher tumor-initiating frequency. Black curves indicate shControl cells and red curves indicate shLHX2 cells. The data are derived from the mouse transplantation experiment detailed in Fig. 6F and significance (p=6.4e^{-12}) was assessed by chi-square test with one degree of freedom (DF). **C** H&E staining and immunohistochemistry of primary tumors in the cerebellum, for pSMAD1/5, SMAD1 and LHX2, after orthotopic xenograft of MB002 and D425 cells (scale bar 20 µm).

Fig. 4A,B

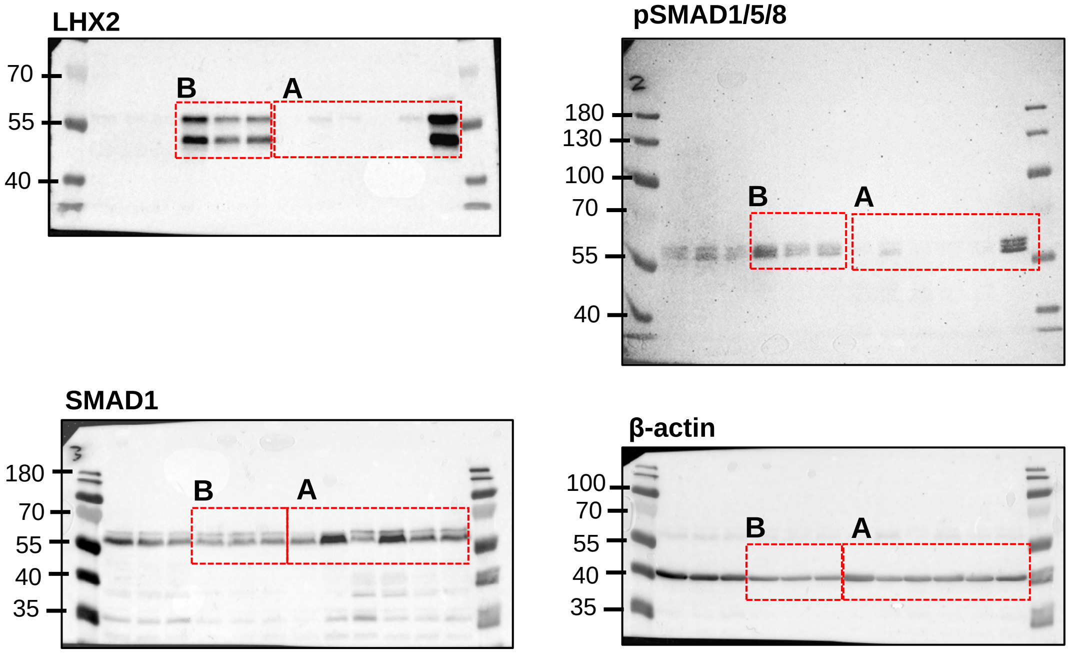

Fig. 4C

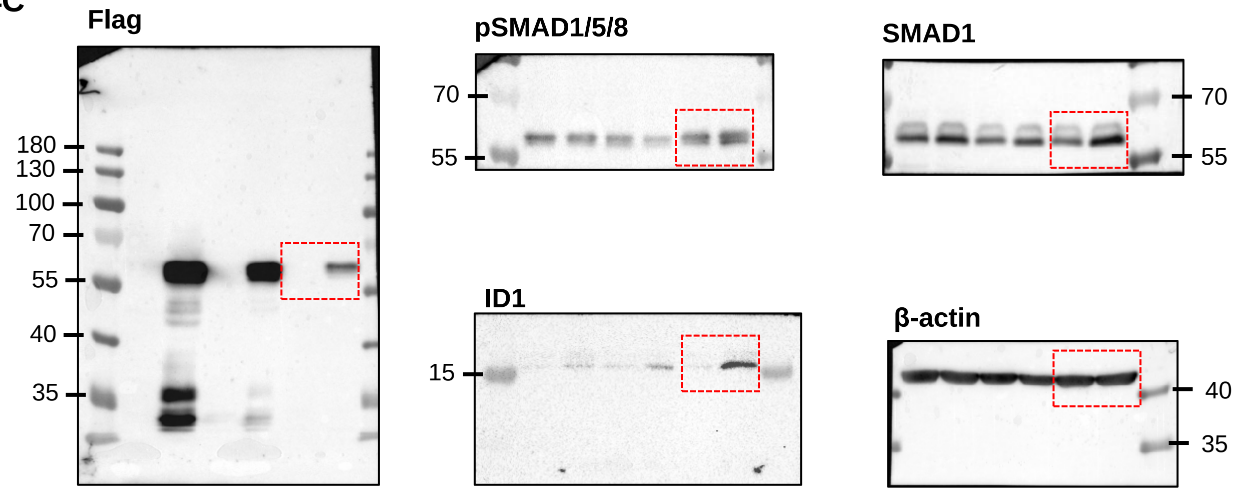

Fig. 4D

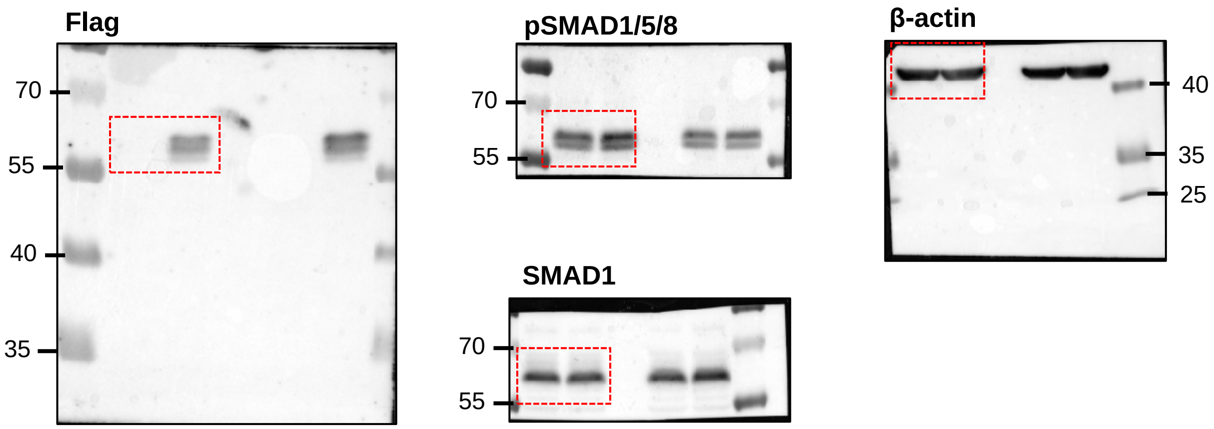

Suppl Fig.S12

Fig. 4G

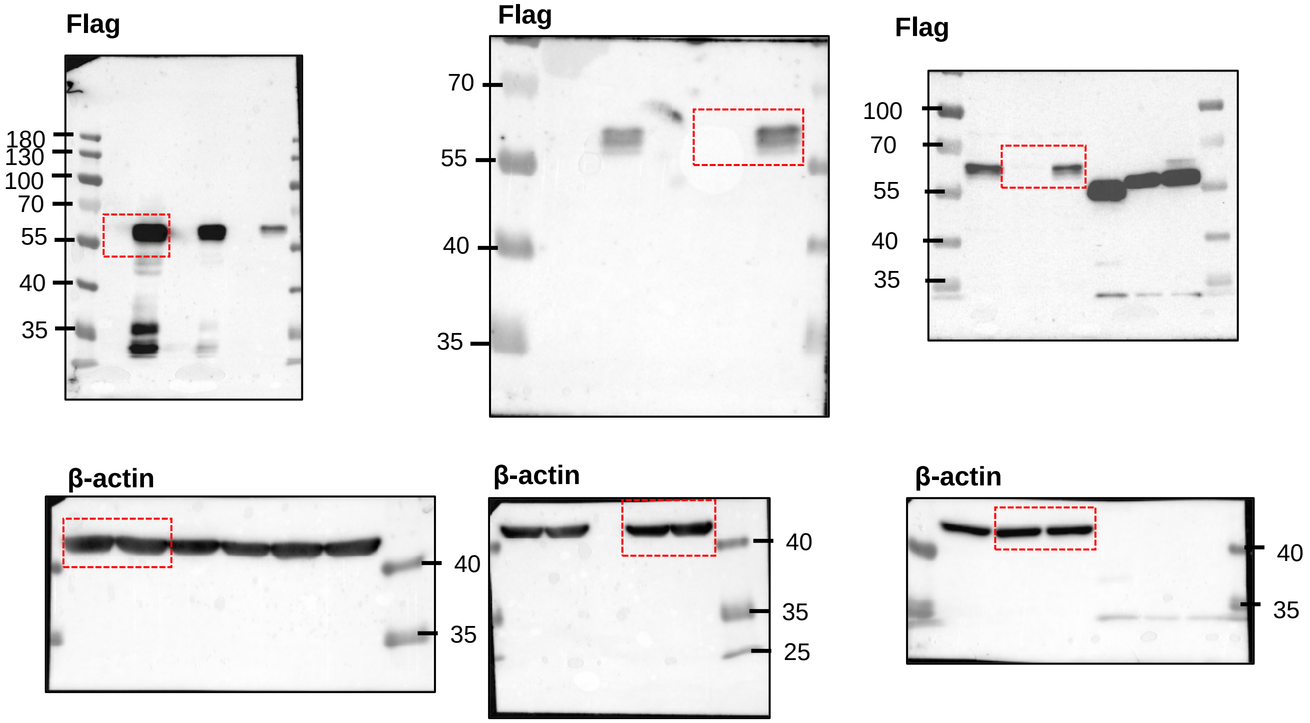

Fig. 6D

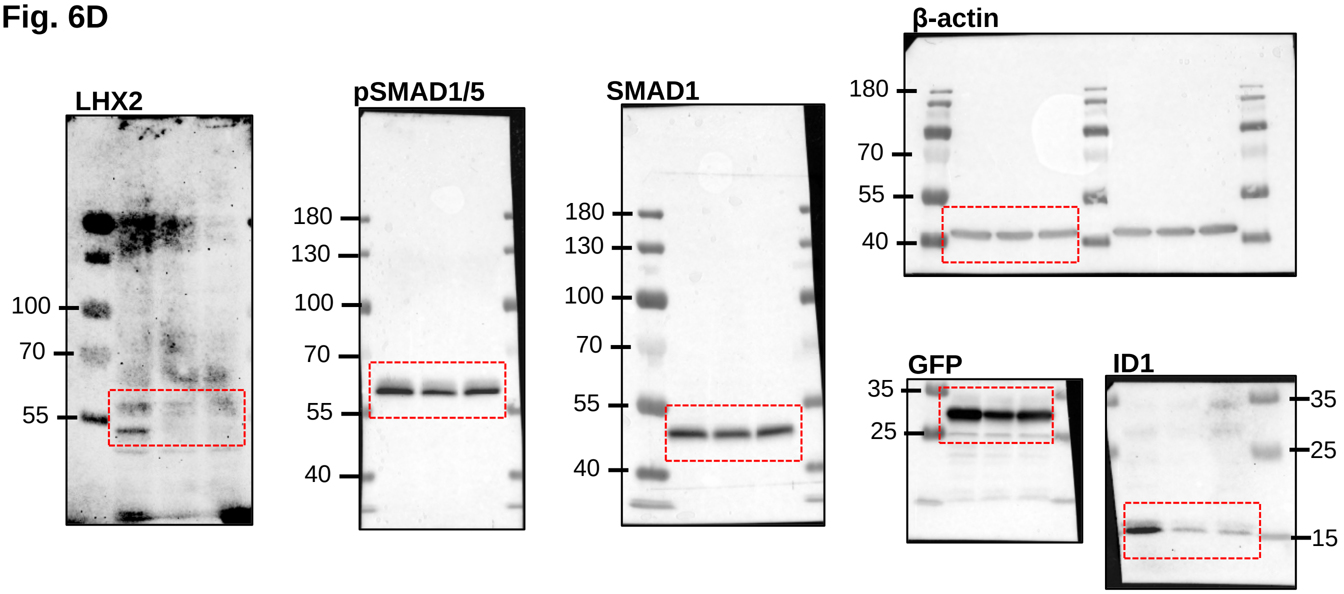

Suppl Fig.S12

Suppl Fig. S6A

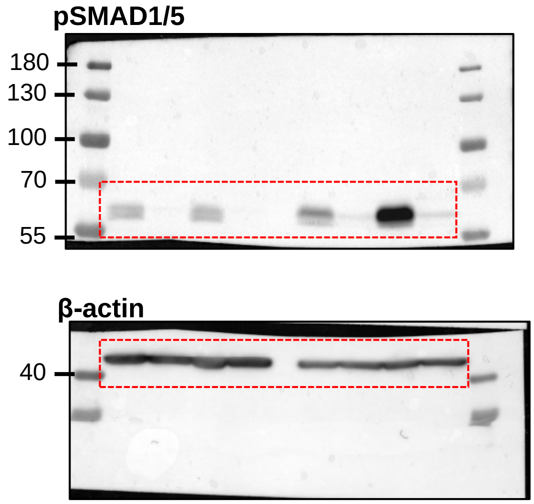

Suppl Fig. S6B

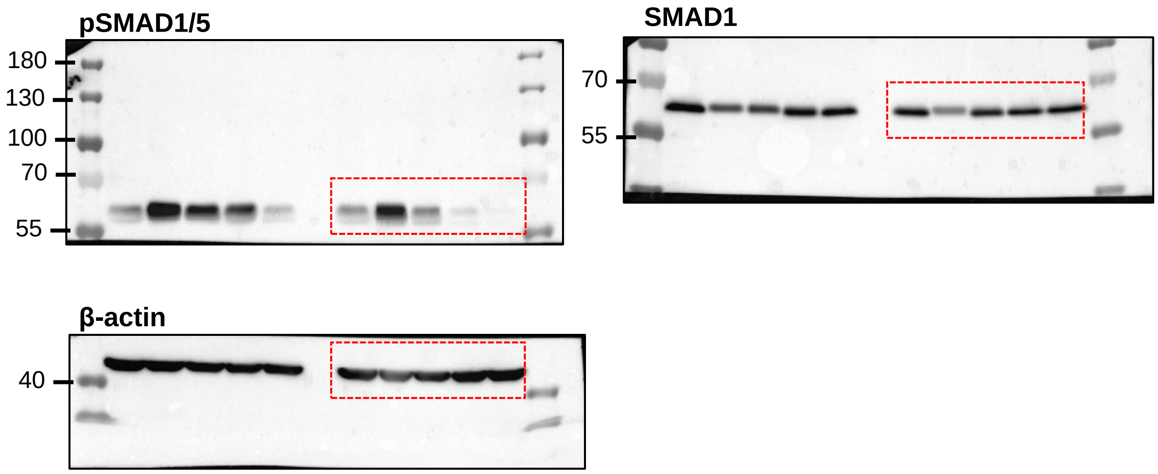

Suppl Fig. S10

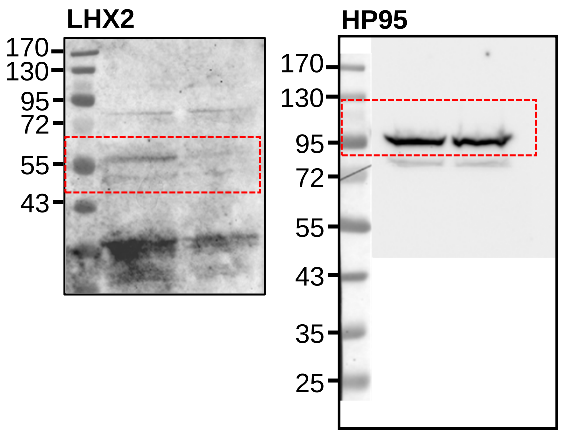

Suppl Fig.S12

Supplementary Fig. S12: Unprocessed immunoblots for the indicated proteins along with molecular mass markers and the corresponding figures. Dotted rectangles demarcate the cropped immunoblots presented in the main or supplementary figures, as indicated in each figure panel.
